# Therapy-Induced Senescence in Cancer Treatment Resistance: Mechanisms, Evidence, and Translational Opportunities

**DOI:** 10.3390/cancers18193084

**Published:** 2026-09-23

**Authors:** Jose-Ramon Blanco, Amancio Carnero

**Affiliations:** 1 Hospital Universitario San Pedro, 26006 Logroño, Spain; 2 Centro de Investigación Biomédica de La Rioja, 26006 Logroño, Spain; 3 Grupo de Investigación en Cuidados y Salud (GRUPAC), Universidad de La Rioja, 26004 Logroño, Spain; 4 Instituto de Biomedicina de Sevilla (IBIS), Hospital Universitario Virgen Del Rocío (HUVR), Consejo Superior de Investigaciones Científicas (CSIC), Universidad de Sevilla, 41013 Sevilla, Spain; 5 CIBER de Cáncer (CIBERONC), Instituto de Salud Carlos III, 28029 Madrid, Spain

**Keywords:** cancer therapy, therapy-induced senescence, cellular senescence, cancer resistance

## Abstract

Cellular senescence is a heterogeneous and context-dependent stress response that can influence cancer treatment response. Therapy-induced senescence (TIS) may initially suppress tumor growth through durable proliferative arrest, whereas persistence of senescent cells may, in specific contexts, contribute to recurrence or resistance through SASP-mediated signaling, metabolic and epigenetic remodeling, cellular plasticity, immune evasion, and changes in the tumor microenvironment. TIS also develops within tissues that may contain pre-existing senescent populations generated by aging, chronic inflammation, environmental stress, metabolic dysfunction, fibrosis, or oncogenic signaling. In this narrative review, we synthesize mechanistic, translational, and clinical evidence linking senescence to resistance across chemotherapy, radiotherapy, targeted therapy, and immunotherapy. We explicitly distinguish experimental mechanisms, clinical associations, and evidence that has or has not undergone prospective validation. Particular attention is given to SASP signaling, cellular plasticity and cancer stemness, senescence escape, metabolic adaptation, immune surveillance, and biomarker limitations. We further evaluate emerging senolytic and senomorphic strategies and the sequential “one–two punch” paradigm, emphasizing differences between preclinical proof-of-concept and clinical evidence. Overall, current data support a context-dependent role for persistent senescence in treatment resistance, but do not establish TIS as a universal or independent cause of therapeutic failure. Clinical translation remains limited by senescence heterogeneity, lack of validated biomarkers, treatment toxicity, uncertainty regarding timing, and insufficient prospective trials.

## Simple Summary

Cancer treatments such as chemotherapy, radiotherapy, targeted therapy, and, in selected settings, immunotherapy can induce cellular senescence, a state characterized by durable cell-cycle withdrawal. Senescence may initially contribute to tumor control, but persistent senescent cells can also modify the tumor microenvironment, alter inflammatory signaling, and support tumor-cell plasticity or survival. Importantly, senescent cells may be present before treatment as a consequence of aging, chronic inflammation, environmental stress, fibrosis, or oncogenic signaling. This review therefore considers therapy-induced senescence (TIS) within its broader tissue context and examines evidence linking senescence-associated phenotypes to treatment response, resistance, and recurrence. We distinguish mechanistic and preclinical evidence from clinical associations and from clinically validated findings, and we discuss the limitations of current senescence biomarkers. Finally, we evaluate senolytic, senomorphic, and sequential “one–two punch” strategies, emphasizing that most therapeutic evidence remains preclinical and that biomarker-guided treatment timing and patient selection remain unresolved.

## 1. Senescence: How Aging, Environmental Stress, and Carcinogenesis Shape a Therapy-Resistant Tumor Microenvironment

### 1.1. Cellular Senescence Beyond Anticancer Therapy

For the purposes of this review, bona fide senescence should be distinguished from transient quiescence, terminal differentiation, apoptosis, immune-cell exhaustion, and other senescence-like phenotypes. No single marker is sufficient to establish senescence across all cell types or tissues. Expression of p16 or p21, increased lysosomal activity, DNA-damage signaling, or selected SASP components should therefore be interpreted as complementary features rather than definitive evidence in isolation. This distinction is especially important in clinical specimens, where tumor, stromal, and immune compartments may display overlapping stress-associated phenotypes.

Cellular senescence is a pleiotropic cell state that can be elicited by a broad spectrum of intrinsic and extrinsic stimuli, including telomere attrition, oncogene activation, oxidative stress, mitochondrial dysfunction, chronic inflammation, genotoxic agents, ultraviolet radiation, tobacco smoke, environmental pollutants, and metabolic dysfunction (Figure 1). Although these diverse stimuli activate distinct upstream signaling pathways, they typically induce durable withdrawal from the cell cycle, accompanied by chromatin reorganization, lysosomal and metabolic remodeling, altered apoptotic susceptibility, and development of the senescence-associated secretory phenotype (SASP) [1,2,3].

Long regarded primarily as an intrinsic tumor-suppressive mechanism that prevents the proliferation of stressed or damaged cells, senescence is now recognized as a context-dependent cell state whose biological effects are determined by the inducing stimulus, cell type, tissue context, duration of the senescent state, and efficiency of immune-mediated clearance. Transient or developmentally programmed senescence can support tissue repair, wound healing, embryonic development, and protection against malignant transformation. By contrast, the persistence and accumulation of senescent cells during aging or sustained environmental stress can maintain inflammatory, fibrotic, and tissue-remodeling programs that facilitate tumor initiation and progression and, in some settings, therapeutic resistance. Accordingly, tumors arising in aged or chronically damaged tissues may encounter a pre-existing reservoir of senescent stromal, epithelial, and immune-cell populations before treatment begins [4,5,6,7,8]. The prevalence, composition, and functional importance of this pretreatment reservoir in human cancers, however, remain incompletely defined.

This conceptual distinction matters clinically: therapy-induced senescence (TIS) does not occur in isolation. Instead, anticancer therapies can act on tissues that already harbor senescent fibroblasts, endothelial cells, epithelial cells, and immune cells, expanding or reprogramming a pre-existing senescent landscape in ways that may influence treatment responses (Figure 1).

### 1.2. Aging and Pre-Existing Senescent Landscape

Physiological aging is accompanied by an increased prevalence of cells displaying senescence-associated features across multiple tissues, although their abundance and phenotype vary among organs, cell types, and individuals. This process is thought to reflect the combined effects of replicative history, telomere dysfunction, genomic and epigenomic damage, oxidative and metabolic stress, mitochondrial dysfunction, and progressively less efficient immune surveillance. Consequently, older adults tend to exhibit higher levels of senescence-associated markers in several stromal and parenchymal compartments. However, the direct quantification of senescent-cell burden in human tissues remains technically challenging. The persistence of these cells may contribute to chronic, low-grade inflammation, often referred to as inflammaging [9].

Age-associated senescent fibroblasts can develop heterogeneous SASP profiles that may include IL-6, IL-8, CCL2, CXCL1, TGF-β, VEGF, matrix metalloproteinases (MMPs), and extracellular matrix (ECM) remodeling proteins. Depending on the tissue and biological context, these mediators can alter tissue architecture and contribute to a permissive niche for malignant transformation [10]. These factors may promote angiogenesis, ECM degradation, epithelial plasticity, and the recruitment of immunosuppressive myeloid populations, thereby creating conditions that support tumor initiation and subsequent resistance to therapy [11,12,13]. These mediators should not, however, be interpreted as a universal SASP signature because SASP composition varies substantially with cell type, senescence inducer, and time since induction.

Aging may affect both the prevalence and the molecular phenotype of senescent cells. Senescence associated with aging cannot be reliably distinguished from acute senescence using a single SASP profile; persistent inflammatory signaling, mitochondrial dysfunction, and inefficient immune clearance are frequent, but not universal, features. Consequently, some older patients may begin anticancer treatment in tissues already shaped by chronic inflammation and senescence-associated remodeling, potentially modifying the effects of therapy-induced senescence [14,15]. The magnitude and clinical relevance of this interaction in human tumors remain incompletely defined and are likely to vary among tumor types and individual patients.

Recent experimental studies have demonstrated that aged stromal fibroblasts can reduce the sensitivity of neighboring tumor cells to chemotherapy by activating context-dependent signaling networks, including signal transducer and activator of transcription 3 (STAT3), nuclear factor κB (NF-κB), and TGF-β pathways. Similarly, senescent endothelial cells may contribute to vascular dysfunction and impaired drug delivery. Age-related immune dysfunction may also reduce cytotoxic activity and the clearance of tumor cells undergoing TIS [16,17]. However, cellular senescence, immune exhaustion, and terminal differentiation represent biologically distinct states and should not be used interchangeably when characterizing immune populations.

Together, these observations provide a mechanistic rationale for investigating whether biological measures of senescence can improve the prediction of treatment response beyond chronological age alone. Chronological age is an imprecise proxy for biological aging and should not be presented as an independent or universal determinant of therapeutic efficacy; functional status, frailty, comorbidities, tumor biology, treatment selection, and pharmacological factors also contribute substantially to clinical outcomes.

### 1.3. Environmental Exposures and Stress-Induced Senescence

In addition to physiological aging, several environmental exposures can induce premature senescence or senescence-like states before clinically detectable tumor development. Relevant exposures include tobacco smoke, ultraviolet (UV) and ionizing radiation, asbestos and crystalline silica; particularly air pollution, including fine particulate matter (PM2.5); selected metals, such as arsenic and cadmium; polycyclic aromatic hydrocarbons and benzene; and chronic alcohol exposure. These agents can induce persistent DNA damage, oxidative stress, mitochondrial dysfunction, telomere instability, and activation of DNA-damage-response pathways involving ataxia-telangiectasia mutated (ATM), ATM- and Rad3-related (ATR), and p53, as well as p16INK4a, ultimately leading to stress-induced premature senescence (SIPS) [18,19]. Unlike replicative senescence, SIPS can develop without extensive replicative exhaustion following acute or chronic environmental exposure and may involve multiple cellular populations within the exposed tissue.

For example, cigarette smoke has been shown in experimental models and human tissue studies to promote SASP in airway epithelial cells, lung fibroblasts, and endothelial cells, which may contribute to chronic obstructive pulmonary disease (COPD), pulmonary fibrosis, and increased lung cancer susceptibility. Likewise, chronic UV radiation can induce senescence-associated changes in dermal fibroblasts and keratinocytes, thereby contributing to a pro-inflammatory cutaneous microenvironment that may facilitate skin carcinogenesis [18]. When environmentally induced senescent cells persist and develop a SASP, their secreted mediators may promote proliferation, angiogenesis, epithelial plasticity, and immune suppression. Consequently, environmental carcinogens may contribute to cancer development not only through mutagenesis but also through long-term remodeling of the tissue microenvironment via senescence [20,21,22,23].

### 1.4. Oncogene-Induced Senescence: From Tumor Suppression to Tumor Promotion

Aberrant activation of the RAS–RAF pathway, including oncogenic BRAF, and, in a context-dependent manner, other oncogenic signaling networks can paradoxically trigger cellular senescence, a phenomenon known as oncogene-induced senescence (OIS). Initially described as a powerful barrier to malignant transformation, OIS can limit the clonal expansion of cells exposed to aberrant mitogenic signaling through mechanisms that frequently involve the p53/p21 and p16INK4a/RB pathways [24,25,26,27].

Histopathological studies have identified combinations of senescence-associated markers in several premalignant lesions, including melanocytic nevi, pancreatic intraepithelial neoplasia, colorectal adenomas, and prostatic intraepithelial neoplasia, supporting OIS as an important early defense against carcinogenesis. However, when OIS cells persist or escape immune-mediated clearance, their SASP may remodel the surrounding microenvironment. Chronic secretion of IL-6, IL-8, TGF-β, VEGF, and MMPs can alter immune-cell recruitment, stimulate angiogenesis, and enhance tissue remodeling, potentially creating conditions that favor malignant progression once additional genetic or epigenetic alterations permit escape from senescence [28,29]. These effects are not uniformly tumor-promoting: in other contexts, OIS-associated secretory programs recruit immune cells that eliminate senescent or premalignant cells. Moreover, progression may reflect either senescence bypass by the affected cells or SASP-mediated support of neighboring non-senescent clones.

Thus, OIS illustrates one of the central paradoxes of senescence biology: a cell-autonomous program that initially restrains tumor initiation can exert tumor-promoting effects when senescent cells persist, immune clearance fails or neighboring cells exploit the resulting secretory milieu.

### 1.5. Chronic Inflammation, Fibrosis, and Senescence

Many cancers arise in chronically inflamed tissues, where repeated injury and repair cycles can generate and favor the persistence of senescent cells. Chronic hepatitis, inflammatory bowel disease, chronic pancreatitis, metabolic dysfunction-associated steatohepatitis (MASH; formerly nonalcoholic steatohepatitis [NASH]), idiopathic pulmonary fibrosis, and chronic gastritis have been associated with increased expression of senescence-related markers in epithelial, endothelial, or stromal compartments.

Persistent SASP production can reinforce inflammatory signaling through NF-κB and Janus kinase/signal transducer and activator of transcription (JAK/STAT) pathways. In parallel, persistent DNA damage, micronuclei, and cytosolic chromatin fragments may activate GMP–AMP synthase–stimulator of interferon genes (cGAS–STING) signaling, thereby contributing to self-sustaining inflammatory circuits that can reinforce senescence in neighboring cells through paracrine senescence. Paracrine senescence can therefore extend the response beyond the initially damaged population and contribute to tissue remodeling and fibrosis [28,30].

Chronically damaged tissues enriched in senescence-associated cells may display increased ECM stiffness, abnormal vascularization, immune suppression, and metabolic features that can limit drug delivery, immune-cell access, or therapeutic responsiveness in specific settings [6,31,32]. Therefore, tumors arising in chronically inflamed or fibrotic organs may be treated within a pre-existing senescence-enriched microenvironment that conditions, rather than predetermines, therapeutic response. Neither inflammation nor fibrosis alone demonstrates cellular senescence; claims of senescence enrichment require convergent evidence from multiple markers.

### 1.6. Pre-Existing Senescence as a Potential Modifier of Therapeutic Response

An emerging hypothesis in cancer biology proposes that TIS develops within a tissue microenvironment that is often already shaped by, and may contain, senescent cells before treatment begins [33]. Rather than representing an isolated biological consequence of chemotherapy or radiotherapy, TIS can therefore be understood as an additional layer superimposed on a pre-existing senescent ecosystem that has progressively evolved during aging, chronic tissue injury, inflammation, metabolic dysfunction, and tumor development itself [7,12,34].

The tumor microenvironment (TME) is a highly heterogeneous and dynamic network composed of stromal fibroblasts, endothelial cells, immune populations, ECM, adipocytes, pericytes, and soluble signaling molecules. Throughout life, repeated exposure to oxidative stress, DNA damage, oncogene activation, mitochondrial dysfunction, telomere attrition, and chronic inflammatory stimuli can promote the accumulation of senescent cells within several of these compartments [35]. Consequently, tumors arising in aged or chronically damaged tissues may encounter senescence-associated tissue remodeling before therapeutic intervention, although the extent of this process in human cancers remains insufficiently defined.

Pre-existing senescence can affect several cellular components of the TME. Some senescent fibroblasts share inflammatory and secretory features with inflammatory cancer-associated fibroblasts (iCAFs), including sustained secretion of ECM-remodeling enzymes, growth factors, chemokines, and pro-inflammatory cytokines that can promote tumor growth, invasion, and therapeutic resistance. However, senescent fibroblasts and iCAFs are not equivalent populations, and these terms should not be used interchangeably without appropriate molecular and functional evidence. Likewise, senescent endothelial cells contribute to vascular dysfunction, increased permeability, impaired drug delivery, and abnormal angiogenic signaling. Aging also reshapes the immune compartments through thymic involution, altered lymphocyte differentiation, reduced natural killer (NK)-cell cytotoxicity, impaired antigen presentation, and the accumulation of regulatory or suppressive immune populations. These changes encompass immunosenescence, exhaustion, and altered differentiation, but only a subset necessarily fulfills criteria for cellular senescence. Collectively, they can weaken effective antitumor immune surveillance [7,35,36].

In parallel, persistent senescent stromal cells can remodel the ECM. Senescent stromal cells may secrete MMPs, matricellular proteins, and context-dependent ECM components, thereby altering matrix composition, tissue stiffness and mechanotransduction and potentially creating physical barriers that limit immune-cell infiltration and therapeutic penetration. Where progressive fibrosis occurs, ECM deposition can enhance the activation of integrin-mediated survival pathways, including focal adhesion kinase (FAK), phosphoinositide 3-kinase/protein kinase B (PI3K/AKT), and Yes-associated protein/transcriptional coactivator with PDZ-binding motif (YAP/TAZ) signaling, which may increase resistance to apoptosis and reduce treatment efficacy [12].

One of the common, although non-universal, characteristics of senescent cells is the SASP, a complex and highly dynamic secretome that may include inflammatory cytokines (IL-6, IL-8, IL-1α, IL-1β), chemokines (CXCL1, CXCL2, CXCL5, CCL2), growth factors (VEGF, HGF, TGF-β, amphiregulin), ECM-remodeling enzymes (MMP-1, MMP-3, MMP-9), lipid mediators, extracellular vesicles, and damage-associated molecular patterns (DAMPs). Persistent SASP production can establish a chronic inflammatory milieu capable of promoting epithelial-to-mesenchymal transition (EMT), angiogenesis, stemness, immune suppression, and tumor cell survival. Conversely, particular SASP programs can enhance immune recognition and clearance, emphasizing that their effects are temporally and contextually dependent. SASP signaling can also induce paracrine senescence, whereby neighboring non-senescent cells undergo secondary senescence, further expanding the senescent compartment and amplifying inflammatory signaling throughout the tissue [37,38]. The production of these factors is coordinated by several interconnected signaling pathways, including persistent DNA damage response signaling, NF-κB, CCAAT/enhancer-binding protein β (C/EBPβ), p38 mitogen-activated protein kinase (p38 MAPK), mechanistic target of rapamycin (mTOR), and the cGAS-STING pathway, which can establish a sustained inflammatory program characteristic of particular senescent states [38,39].

Following chemotherapy, radiotherapy, targeted therapies, or certain immunotherapies, this pre-existing senescent landscape may expand or change in composition. DNA-damaging therapies induce senescence not only in malignant cells but also in fibroblasts, endothelial cells, and other stromal populations; immune cells may additionally develop senescence-like or dysfunctional states. Consequently, the overall burden of senescent cells can increase, augmenting SASP production and reinforcing pro-survival signaling networks. Rather than initiating an entirely new biological program, anticancer treatments may amplify biological processes that are already active within the aged or chronically damaged TME [40,41].

In selected experimental models, an increased senescent-cell burden has been associated with reduced responsiveness to anticancer therapy. However, a generalized clinical relationship across tumor types, treatment modalities, and older patient populations has not been established. Experimental models have shown that aged tissues can exhibit enhanced inflammatory signaling, impaired immune-mediated clearance of senescent cells, persistent activation of NF-κB and STAT3 pathways, increased TGF-β signaling, and elevated expression of anti-apoptotic proteins such as members of the BCL-2 family [12,42,43].

Therapy resistance emerges not simply from the presence of senescent cells but from their interactions within the broader tissue ecosystem. SASP-derived IL-6 and IL-8 can activate STAT3-dependent transcriptional programs that enhance cancer stem cell maintenance and epithelial plasticity. TGF-β may promote EMT, immune exclusion, and fibrosis, while VEGF and angiogenic mediators can remodel tumor vasculature. Matrix remodeling enzymes may facilitate invasion and metastatic dissemination, whereas immunosuppressive cytokines can impair cytotoxic lymphocyte recruitment and function. Collectively, when sustained, these interconnected pathways can create a self-reinforcing microenvironment in which cancer cells are increasingly protected from both cytotoxic injury and immune elimination.

The concept of total senescence burden offers a broader conceptual framework than TIS alone but does not yet constitute a validated clinical metric (Figure 2). The biological impact of treatment may depend not only on the number of cells rendered senescent during therapy but also on the pre-existing accumulation of senescent stromal and immune cells established through decades of physiological aging, environmental exposures, chronic inflammation, fibrosis, metabolic disease, and oncogenic stress. In this model, TIS may function as an amplifier of a pre-existing dysfunctional ecosystem, an initiating remodeling event, or a transient tumor-suppressive response, depending on the tissue, treatment, and temporal context. Clinical evaluation of this model will require longitudinal, spatially resolved, and cell-type-specific measurements that distinguish senescent-cell abundance from SASP activity and other forms of cellular dysfunction.

This perspective has important therapeutic implications. Senolytic drugs, senomorphic agents, and immune-based clearance strategies should therefore be evaluated against both treatment-induced and pre-existing senescent compartments, rather than against tumour cells undergoing TIS alone. Whether broad reduction in the senescent-cell burden is preferable to selective targeting of particular cellular compartments remains an open question. Rational combination strategies will need to consider cellular identity, spatial distribution, and therapeutic timing, as indiscriminate senescent-cell depletion could also remove transiently beneficial populations. Such strategies could integrate senescence-targeting interventions with conventional anticancer treatments to restore immune surveillance, restrain chronic SASP signaling, improve treatment responsiveness, and reduce the risk of recurrence.

Taken together, these observations motivate a working model in which therapeutic resistance can emerge from multiple senescence-inducing processes acting sequentially and concurrently during tumor evolution and treatment. Aging, chronic tissue damage, metabolic dysfunction, inflammation, oncogenic signaling, and anticancer therapies may collectively generate a progressively senescent tumor ecosystem that favors residual disease and limits treatment efficacy. Considering pre-existing senescence as a potential modifier of treatment outcome shifts attention from TIS as an isolated phenomenon toward the cell-type-resolved senescent landscape that may contribute to cancer progression and resistance.

## 2. Therapy-Induced Senescence: A Context-Dependent Response to Anticancer Treatment

One of the major advances in cancer biology over the past two decades has been the recognition that cellular senescence is not only induced by endogenous stresses such as telomere erosion or oncogenic activation, but can also emerge as a context-dependent response to anticancer therapy. Diverse treatment modalities, including chemotherapy, radiotherapy, cyclin-dependent kinase 4/6 (CDK4/6) inhibitors, poly(ADP-ribose) polymerase (PARP) inhibitors, and selected kinase inhibitors, can elicit senescence or senescence-like phenotypes through genotoxic or cytostatic mechanisms in malignant cells and, depending on tissue exposure, non-malignant compartments. TIS has emerged as a potential modifier of therapeutic outcome because it can initially suppress tumor growth while later creating, in particular contexts, conditions that favor disease recurrence and treatment resistance [44].

Unlike apoptosis, which eliminates damaged cells through programmed cell death, TIS allows affected cells to remain metabolically active despite a durable withdrawal from the cell cycle. In malignant cells, however, the durability of this arrest cannot always be assumed. Senescent tumor cells retain biosynthetic activity, may undergo extensive metabolic and epigenetic remodeling, and can develop a heterogeneous SASP. Consequently, the biological effects of anticancer therapy extend far beyond the direct elimination of tumor cells and include potentially persistent alterations in the TME that may influence neighboring malignant cells, stromal fibroblasts, endothelial cells, and immune populations.

The induction of TIS is frequently associated with persistent activation of the DNA damage response (DDR) following genotoxic therapy. DNA double-strand breaks generated by ionizing radiation or genotoxic chemotherapeutic agents predominantly engage ATM–CHK2 signaling, whereas replication stress and regions of replication protein A (RPA)-coated single-stranded DNA preferentially activate ATR–CHK1; substantial crosstalk occurs between these pathways. Both signaling axes can promote p53 stabilization. Activated p53 induces transcription of the cyclin-dependent kinase inhibitor p21WAF1, resulting in inhibition of cyclin-CDK complexes and cell-cycle arrest. In parallel, sustained cellular stress can promote activation of the p16INK4a-RB pathway, which reinforces durable proliferative arrest through maintenance of RB in its hypophosphorylated state. This pathway is not universal, particularly in malignant cells harboring CDKN2A or RB-pathway alterations. Although these pathways overlap with those involved in replicative and oncogene-induced senescence, TIS does not constitute a unique molecular program: it is defined by its therapeutic trigger, whereas its molecular architecture depends on treatment modality, dose, cell lineage, and genetic background [3,39,45]. Persistent DNA lesions or incomplete repair can maintain DDR signaling even after the therapeutic exposure has ended.

In addition to DNA damage, several non-genotoxic mechanisms contribute to TIS. Many anticancer agents induce production of reactive oxygen species (ROS), mitochondrial dysfunction, endoplasmic reticulum stress, and disturbances in cellular metabolism, all of which can engage senescence signaling pathways [38]. Persistent oxidative stress can amplify DDR signaling, while mitochondrial dysfunction may promote activation of NF-κB and C/EBPβ, two transcription factors that orchestrate SASP expression [11,46]. Cytoplasmic chromatin fragments generated following nuclear-envelope and chromatin disruption can activate the cGAS-STING pathway, providing an additional mechanism linking genomic instability to inflammatory SASP production [47]. Depending on cellular and temporal context, cGAS–STING signaling may also enhance type I interferon responses and immune-mediated clearance. These interconnected networks can sustain senescence-associated inflammation after the initiating insult has subsided; however, the SASP is dynamic rather than stable and is neither universal nor uniformly pro-inflammatory.

TIS is not restricted to cancer cells. Stromal fibroblasts, endothelial cells, and other non-malignant stromal populations exposed to chemotherapy or radiotherapy may also undergo senescence. Some immune populations can acquire senescence-like phenotypes, although these must be distinguished from exhaustion, anergy, and terminal differentiation. Senescent stromal cells can produce abundant SASP factors that reshape the TME, promote ECM remodeling, and influence the behavior of surviving tumor cells [12]. Consequently, therapeutic responses are better understood as tissue-wide phenomena involving complex interactions between malignant and non-malignant compartments rather than exclusively cell-autonomous responses confined to cancer cells.

Initially, TIS was considered a desirable therapeutic endpoint because permanent proliferative arrest was thought to prevent tumor regrowth. Indeed, numerous experimental models have shown that senescence induction can correlate with reduced tumor burden and improved short-term treatment responses. However, increasing evidence indicates that senescence is considerably more dynamic than originally appreciated. Senescent cells may persist for prolonged periods following treatment, escape immune-mediated clearance, or even regain proliferative capacity under specific conditions. Such observations should be interpreted carefully because apparent escape may reflect true reversal of senescence, selection of non-senescent persister cells, or incomplete establishment of the senescent state. Moreover, persistent SASP production can establish a chronically inflammatory microenvironment that promotes EMT, cancer stem cell plasticity, angiogenesis, ECM remodeling, and immune suppression. Conversely, particular SASP programs enhance tumor immunogenicity and recruit immune cells that eliminate senescent cells. These discoveries have established TIS as a double-edged biological response that can contribute to therapeutic failure [48].

The realization that therapies themselves can generate populations of persistent senescent cells has important clinical implications. Rather than necessarily representing a terminal treatment endpoint, TIS may function as a transitional state that shapes subsequent tumor evolution and treatment response. Understanding the molecular mechanisms governing the induction, maintenance, and eventual fate of cells undergoing TIS is therefore important for clarifying their potential contribution to resistance to chemotherapy, radiotherapy, targeted therapies, and, potentially, immunotherapy. These concepts have also provided a preclinical rationale for strategies aimed at selectively eliminating senescent cells after conventional treatment, a concept known as the “one–two punch” approach, which combines senescence-inducing therapies with subsequent administration of senolytic agents [45,49].

## 3. Mechanisms Linking Therapy-Induced Senescence to Cancer Therapy Resistance

The mechanisms discussed below should be interpreted as context-dependent rather than as evidence that TIS invariably produces treatment resistance. Experimental studies provide the strongest support for specific causal pathways, whereas patient studies frequently identify associations between senescence-associated features and clinical outcomes. The distinction is maintained throughout the review.

### 3.1. The Senescence-Associated Secretory Phenotype as a Context-Dependent Mediator of Therapy Resistance

Although TIS can initially constrain tumor growth by limiting the proliferation of damaged cells, persistent cells undergoing TIS can subsequently alter the TME through the SASP (Table 1). The SASP is now recognized as one of several mechanisms linking TIS to cancer recurrence and resistance, acting through both autocrine and paracrine signaling pathways. Rather than representing a static collection of inflammatory mediators, the SASP is a highly dynamic secretome whose composition varies according to the inducing stimulus, cell type, tissue context, and duration of senescence [3,11,50]. Accordingly, the SASP should not be interpreted as a single molecular signature or as an obligatory feature of every senescent cell.

In appropriate physiological and tumor contexts, selected SASP components can support beneficial biological responses [11,51]. Autocrine signaling can reinforce cell-cycle arrest, whereas paracrine signals can recruit or activate NK cells, macrophages, and T lymphocytes that eliminate senescent cells through immune surveillance [6,52,53]. These processes can contribute to tissue repair and reinforce the tumor-suppressive function of senescence [54]. However, when senescent cells persist following anticancer therapy because of inefficient immune clearance or activation of senescent-cell survival programs, prolonged SASP secretion may become maladaptive [12,55]. Chronic exposure of the TME to particular combinations of inflammatory and matrix-modifying mediators can promote tissue remodeling, angiogenesis, fibrosis, immune dysfunction, and the survival of residual malignant cells, thereby creating favorable conditions for disease recurrence [56,57].

One of the most extensively studied components of the SASP is IL-6, which can activate the JAK/STAT3 signaling pathway in neighboring receptor-competent tumor cells [58]. Persistent STAT3 activation can enhance proliferation, inhibit apoptosis, promote EMT, and support the acquisition of stem cell-like characteristics, thereby contributing, in selected models, to resistance to chemotherapy and targeted therapies [59,60]. Similarly, IL-8 stimulates CXCR1/CXCR2 signaling, promoting migration, invasion, angiogenesis, and expansion of therapy-resistant cancer stem cell populations in specific tumor contexts [37]. Elevated circulating or intratumoral levels of IL-6 and IL-8 have been correlated with poor prognosis and reduced response to treatment in multiple malignancies, including breast, colorectal, pancreatic, lung, and ovarian cancers [61].

TGF-β, a context-dependent SASP component, may contribute to therapeutic resistance by inducing EMT, promoting ECM deposition, and suppressing antitumor immune responses [62,63]. TGF-β remains pleiotropic and can exert tumor-suppressive effects in premalignant or early-stage epithelial cells; its biological effect depends on cellular genotype and disease stage. Increased matrix stiffness and fibrosis reduce drug penetration into some solid tumors while simultaneously activating mechanotransduction pathways, including YAP/TAZ signaling, which may enhance tumor cell survival [64]. MMPs secreted by senescent stromal cells can remodel the ECM, facilitate tumor invasion, and release matrix-bound growth factors that may stimulate malignant progression. However, their net effects are tissue-dependent because matrix degradation can also alter immune-cell access and tissue repair.

Beyond soluble factors, proteomic studies indicate that senescence can alter both the release and molecular cargo of extracellular vesicles (EVs) containing proteins, lipids, microRNAs, long non-coding RNAs, and DNA fragments capable of modifying the phenotype of neighboring cells [65,66]. In experimental systems, senescence-associated EVs can alter signaling pathways involved in apoptosis, DNA repair, metabolism, and stemness [67]. For example, senescence-associated EVs have been shown to activate proliferative signaling pathways in recipient tumor cells while simultaneously enhancing resistance to DNA-damaging agents [68]. These observations suggest that extracellular vesicle-mediated communication represents an additional layer of SASP-mediated intercellular signaling that extends the influence of senescent cells beyond the immediate microenvironment.

The composition of the SASP is not static but evolves over time [50,51]. Some experimental models display an early TGF-β-rich secretory program followed by a later pro-inflammatory SASP, whereas other models follow different temporal trajectories. Transient senescence followed by efficient immune clearance is often associated with tissue repair or tumor suppression, whereas persistent senescent cells can maintain maladaptive inflammatory and matrix-remodeling signals [2,54]. This temporal evolution provides a plausible mechanistic hypothesis linking favorable short-term responses to subsequent recurrence; however, direct clinical evidence connecting longitudinal SASP trajectories with recurrence months or years after treatment remains limited. The persistence of senescent cells is therefore likely to be one of several determinants of whether senescence remains tumor suppressive or becomes tumor promoting.

Collectively, current evidence indicates that the SASP represents a major interface through which cells undergoing TIS communicate with their surrounding TME. Rather than acting exclusively as arrested cells, senescent tumor and stromal cells can participate in inflammatory, metabolic, and structural remodeling that supports the survival and adaptation of residual malignant cells. Other senescence-associated mechanisms, including altered antigen presentation, cell-surface ligands, metabolic competition, and direct ECM interactions, may operate independently of the SASP. Consequently, targeting SASP production or selectively eliminating persistent senescent cells has emerged as an attractive therapeutic strategy aimed at preventing treatment resistance without compromising the initial antitumor effects of senescence (Figure 3 and Table 1).

**Table 1:** Summary of SASP factors associated with signaling pathways, effects, and their association with therapy resistance.

Cancer Type	Therapy	Senescence-Associated Features	Clinical Association	Evidence Level	Key Limitation	Refs
Breast	Anthracyclines, taxanes; CDK4/6 inhibitors	p21, p16, IL-6, IL-8	Residual disease, relapse	Clinical association	Markers are not specific for bona fide senescence; causality is not established.	[43,48]
NSCLC	Platinum; EGFR inhibitors	p21, γH2AX, SASP	Drug resistance	Clinical/mechanistic association	Overlap with treatment-stress and resistant persister states.	[39]
Colorectal	Oxaliplatin, irinotecan	SASP, IL-8	Tumor recurrence	Clinical association	SASP components are context-dependent and not unique senescence markers.	[3]
Pancreatic	Gemcitabine, radiation	SASP, CXCLs	Stromal remodeling	Preclinical/clinical association	Tumor-cell and stromal senescence may be difficult to separate in tissue samples.	[31]
Melanoma	BRAF/MEK inhibitors	Senescence-associated transcriptional signature	Adaptive resistance	Mechanistic/preclinical	Transcriptional signatures may capture heterogeneous persister states.	[57]
Prostate	Radiotherapy	p16, TGF-β	Fibrosis and recurrence	Clinical association	Fibrosis and inflammatory signaling are not specific to senescence.	[69]
Ovarian	Platinum	IL-6, IL-8	Chemoresistance	Clinical/preclinical association	Longitudinal validation and senescence-specific biomarker confirmation remain limited.	[70,71]

### 3.2. Therapy-Induced Senescence Promotes Cellular Plasticity and Cancer Stemness

A major conceptual advance in the field of TIS is the recognition that senescence not only arrests proliferation but can also alter the phenotype of neighboring cells. Through sustained secretion of SASP components, senescent cells can establish a microenvironment that promotes cellular plasticity, dedifferentiation, and the acquisition of stem cell-like properties by residual cancer cells. This evidence challenges a purely tumor-suppressive view of senescence and instead supports a context-dependent role in tumor evolution and long-term therapeutic resistance [12,57].

Cancer stem cells (CSCs) denote functionally defined tumor-cell states characterized by self-renewal, tumor-initiating capacity, and the ability to regenerate tumor heterogeneity [72,73]. Unlike the bulk of proliferating tumor cells, CSCs may exhibit enhanced DNA repair, elevated antioxidant defenses, metabolic flexibility, and intrinsic resistance to apoptosis. Thus, although chemotherapy and radiotherapy can efficiently debulk tumors, CSCs that survive treatment may seed relapse and metastatic dissemination. Increasing evidence indicates that TIS may contribute to this process by generating de novo stem-like states in addition to selecting pre-existing CSCs [57].

The SASP comprises factors that can induce cellular reprogramming [57]. Among these, IL-6 can promote stemness through the JAK/STAT3 signaling pathway [59]. Sustained STAT3 signaling can increase the expression of stemness-associated factors, including SOX2, NANOG, OCT4, KLF4, and c-MYC, in a context-dependent manner, thereby favoring the dedifferentiation of non-stem cancer cells into cells with stem cell-like characteristics [59]. Similarly, IL-8 signaling through CXCR1 and CXCR2 can support self-renewal, tumor sphere formation, migration, and resistance to chemotherapy in multiple tumor types, including breast, pancreatic, colorectal, ovarian, and lung cancers [74]. These cytokines may act in concert with additional SASP components such as CXCL1, CXCL5, and CCL2 to reinforce inflammatory circuits that sustain stemness [74].

Beyond inflammatory cytokines, TIS can engage several developmental signaling pathways that regulate cell fate determination [57]. WNT/β-catenin, NOTCH, Hedgehog, Hippo-YAP/TAZ, and TGF-β signaling have all been implicated in senescence-associated cellular reprogramming. Depending on the cellular context, activation of these pathways can increase transcriptional plasticity, allowing differentiated tumor cells to reacquire progenitor-like characteristics [48,75]. This phenomenon is particularly relevant following exposure to chemotherapy or targeted therapies, when surviving tumor cells can undergo extensive transcriptional remodeling without necessarily acquiring new driver mutations [75]. Therapy resistance may therefore arise from interactions between microenvironment-driven phenotypic adaptation and Darwinian selection of resistant clones.

Milanovic and colleagues [57] provided direct evidence for a related but mechanistically distinct process. Using lymphoma and leukemia models, they showed that cells entering TIS acquired a largely cell-autonomous, WNT-dependent stemness program. After release from the arrest, previously senescent cells displayed enhanced clonogenic and tumor-initiating capacity. The study further identified permissive chromatin remodeling at WNT- and stemness-associated promoters and showed that genetic or pharmacological inhibition of WNT–β-catenin signaling abrogated the excess clonogenicity without preventing TIS. These findings establish that TIS can generate latent stem-like potential within senescent cancer cells themselves, a mechanism distinct from SASP-mediated reprogramming of neighboring cells [57].

Epigenetic remodeling constitutes another important mechanism linking senescence to stemness [8]. Senescent cells undergo profound alterations in chromatin organization, including the context-dependent formation of senescence-associated heterochromatin foci (SAHF), redistribution of histone modifications, changes in DNA methylation, and remodeling of enhancer landscapes [30,76]. Although these epigenetic alterations can help stabilize growth arrest within senescent cells themselves, SASP-mediated signaling can also trigger epigenetic reprogramming in neighboring proliferating cells. Such remodeling may increase the accessibility of developmental, regenerative, and epithelial plasticity programs, thereby favoring the emergence of therapy-resistant phenotypes.

The relationship between senescence and EMT further reinforces cellular plasticity. Numerous SASP components, particularly TGF-β, IL-6, and IL-8, can induce expression of EMT-associated transcription factors such as SNAIL, SLUG, TWIST, and ZEB1/2 [75,77,78]. EMT can enhance migratory and invasive properties and is frequently associated with stem cell-like traits, resistance to apoptosis, and metabolic adaptations that facilitate survival under therapeutic stress [2,77]. EMT and stemness should therefore be viewed as interrelated, although not equivalent, manifestations of senescence-driven phenotypic plasticity rather than independent biological processes.

The effects of TIS are not confined to malignant epithelial cells. TIS can also arise in stromal and endothelial cells and alter immune-cell phenotypes within the TME [12]. Senescent stromal cells can produce ECM proteins, growth factors, and inflammatory mediators that establish niches that support CSC survival and self-renewal. This reciprocal interaction between senescent stromal cells and therapy-resistant tumor cells may amplify intratumoral heterogeneity and favor long-term disease persistence after an initially effective treatment [11,57].

Collectively, the evidence supports at least two nonexclusive routes by which TIS may promote resistance: cell-autonomous acquisition of latent stemness by senescent cancer cells and paracrine, SASP-mediated reprogramming of neighboring tumor and stromal cells [57,79] (Figure 4). Persistent senescent cells may therefore function not only as arrested cells but also as regulators of tissue remodeling and phenotypic diversification [12]. Defining the relative contribution and temporal dynamics of these mechanisms will be essential for determining whether recurrence can be prevented by eliminating senescent cells, suppressing detrimental SASP signaling, blocking reprogramming pathways, or targeting the stem-like populations that emerge after therapy.

### 3.3. Escape from Therapy-Induced Senescence: A Source of Tumor Recurrence and Acquired Resistance

Cellular senescence is classically defined as a stable cell-cycle arrest, maintained in part by the p53–p21 and p16INK4a-RB pathways and reinforced by epigenetic remodeling. Nevertheless, longitudinal studies have shown that some therapy-treated cancer cells displaying multiple senescence features can subsequently resume proliferation. Such observations support the existence of senescence escape in defined experimental settings, but do not establish that TIS is generally reversible. Demonstrating bona fide escape requires evidence that the same cells first entered an established senescent state and later re-entered the cell cycle; population-level coexistence of senescence and proliferation markers is insufficient [3,48].

The stability of TIS appears to depend on both the genetic background of the tumor and the nature of the senescence-inducing stimulus [57]. Cancer cells frequently harbor mutations affecting TP53, CDKN2A, RB1, PTEN, or other regulators of cell-cycle control, which may reduce the stability of senescence-associated growth arrest. Consequently, while many cells remain permanently arrested, a small fraction may resume proliferation after checkpoint erosion, pathway rewiring, or loss of senescence-maintenance signals. Cells that escape TIS can display increased genomic instability, phenotypic plasticity, and resistance to subsequent treatment [80,81].

One proposed mechanism of senescence escape involves progressive attenuation of persistent DDR signaling [57,82,83]. During the establishment of TIS, sustained activation of ATM, ATR, CHK1, CHK2, and p53 maintains high expression of p21, thereby preventing cell-cycle progression. Over time, however, surviving tumor cells may gradually resolve portions of the DDR through DNA repair, checkpoint adaptation, or compensatory activation of survival pathways such as PI3K/AKT, MAPK, and mTOR signaling. Declining p21 expression or functional inactivation of RB may subsequently permit reactivation of cyclin-dependent kinases, allowing selected cells to resume proliferation despite persistent genomic abnormalities [84,85]. Escaping cells may retain chromosomal rearrangements and DNA lesions acquired during therapy, increasing genetic diversity within the recurrent tumor.

Epigenetic plasticity represents another potential determinant of senescence reversibility. Although senescence is associated with extensive chromatin remodeling, including context-dependent formation of SAHF, redistribution of histone modifications, and large-scale transcriptional reprogramming, these changes are not necessarily irreversible in malignant cells. Chromatin reconfiguration may restore proliferation-associated transcription while preserving or activating stemness and EMT programs. Senescence escape should therefore be considered a product of interacting epigenetic and genetic changes, rather than being attributed exclusively to either mechanism [57,86,87].

Exposure to chemotherapy or radiotherapy frequently induces extensive DNA damage that promotes mitotic failure, endoreduplication, or cytokinesis defects, generating highly enlarged polyploid cells that initially exhibit many features of senescence, including senescence-associated β-galactosidase (SA-β-gal) activity, persistent DDR signaling, and expression of p21 and p16 [88]. However, polyploidy and senescence are overlapping but non-equivalent states, and expression of individual senescence markers does not establish that a polyploid giant cancer cell (PGCC) has undergone bona fide senescence. In several experimental models, surviving PGCCs have generated proliferative progeny through depolyploidization, budding, or atypical divisions; this progeny can exhibit tumor-initiating capacity and treatment resistance. The frequency and clinical relevance of these events remain incompletely defined [88].

These observations have led to the concept that senescence and polyploidization represent interconnected adaptive responses rather than independent cellular programs. Polyploid cells may function as transient reservoirs that preserve tumor cell viability during periods of intense therapeutic stress while simultaneously facilitating genomic reorganization. Upon re-entry into the proliferative cycle, their progeny may exhibit extensive chromosomal instability, increased mutational burden, and marked phenotypic heterogeneity, thereby accelerating Darwinian selection of highly resistant clones. This evolutionary perspective suggests that therapy itself may inadvertently generate cellular diversity from which resistant populations subsequently emerge [89,90].

Senescence escape is further facilitated by the TME. Persistent SASP signaling provides abundant growth factors, inflammatory cytokines, and ECM-remodeling enzymes that support survival of arrested cells during the transition toward renewed proliferation. IL-6 and IL-8 activate STAT3-dependent transcriptional programs that enhance cell survival and self-renewal, whereas HGF, EGF, and TGF-β stimulate proliferative and migratory signaling pathways in neighboring cells. These paracrine effects can cooperate with cell-autonomous checkpoint failure, although direct evidence that individual SASP components drive escape from an established senescent state remains model-dependent [11,50].

The immune system also plays a decisive role in determining whether senescent cells remain arrested or contribute to recurrence. Under physiological conditions, NK cells, macrophages, and cytotoxic T lymphocytes recognize and eliminate senescent cells through a process termed senescence surveillance. However, anticancer therapies frequently induce lymphopenia, alter myeloid cell polarization, and promote immune exhaustion, thereby reducing the efficiency of senescent cell clearance. Persistent senescent cells that escape immune surveillance continue to secrete SASP factors and remain available for eventual reactivation. Defective surveillance may therefore permit senescent or senescence-like cells to persist within residual disease. Whether this persistence directly enables later cell-cycle re-entry and relapse in patients remains to be established [91,92].

Clinical evidence for senescence escape remains indirect. Residual tumors after therapy may contain p21-, p16INK4a-, or SA-β-gal–positive cells together with Ki-67–positive populations, and recurrent tumors may coexpress senescence-associated, inflammatory, stemness, and DNA-repair programs. However, none of these markers is specific for senescence, and their coexistence does not demonstrate that previously senescent cells generated the recurrent tumor. Because lineage tracing is generally not feasible in patients, a direct causal contribution of senescence escape to human recurrence has not yet been established [57,93].

Collectively, these observations have transformed the understanding of TIS from a static endpoint into a dynamic adaptive state capable of influencing long-term tumor evolution. The ability of a subset of senescent cells to regain proliferative capacity, generate genetically diverse progeny, and acquire stem cell-like characteristics provides a compelling explanation for disease recurrence following initially successful treatment. These findings motivate “one–two punch” strategies in which a pro-senescence treatment is followed by senolytic or immune-mediated clearance. Whether such approaches prevent escape or improve clinical outcomes requires prospective evaluation [94,95].

### 3.4. Metabolic Reprogramming of Therapy-Induced Senescent Cells Supports Tumor Survival and Drug Resistance

Metabolic adaptation is a prominent but heterogeneous feature of TIS and represents one of the principal mechanisms enabling senescent cells to persist following anticancer treatment. Although senescent cells typically enter a durable arrest from the cell cycle, they remain highly metabolically active, requiring substantial amounts of energy to sustain protein synthesis, organelle turnover, chromatin remodeling, and continuous production of the SASP. Rather than entering a metabolically quiescent state, senescent cells undergo extensive metabolic reprogramming that promotes long-term survival under conditions of therapeutic stress while simultaneously modifying the metabolic landscape of the TME [3,38].

One of the most frequently reported metabolic alterations observed during TIS is profound mitochondrial remodeling. Senescent cells typically accumulate enlarged and dysfunctional mitochondria characterized by altered morphology, impaired mitochondrial quality control, and increased production of ROS. Although excessive ROS generation contributes to the initiation of senescence by amplifying DDR signaling, persistent mitochondrial dysfunction subsequently becomes a defining feature of the senescent phenotype. The relationship between mitochondrial dysfunction and the SASP is nevertheless context-dependent: mitochondrial dysfunction–associated senescence can produce a secretory program distinct from the canonical IL-1-dependent inflammatory SASP. Mitochondrial dysfunction should therefore not be assumed to uniformly amplify the same inflammatory pathways across senescence models [50].

Mitochondrial dysfunction is accompanied by substantial changes in cellular bioenergetics. Depending on tumor type and therapeutic context, senescent cells exhibit increased reliance on oxidative phosphorylation (OXPHOS), enhanced glycolytic flux, or a hybrid metabolic phenotype capable of switching between energy sources according to nutrient availability [50,96]. Such heterogeneity probably reflects differences in lineage, senescence trigger, and the degree of mitochondrial impairment. Increased mitochondrial respiration has been reported in several models of TIS and may provide ATP for protein synthesis and SASP production, whereas increased glycolysis can sustain ATP generation and biosynthetic demands. Thus, senescent cells redirect metabolic resources from proliferation toward cellular maintenance and intercellular signaling.

Autophagy also plays a pivotal role in metabolic adaptation during TIS [97,98]. Its role is context-dependent: autophagic and lysosomal pathways can remove damaged components, recycle metabolites, and support SASP production, but autophagic flux may also be impaired in some senescent states. Accordingly, inhibition of autophagy can reduce senescent-cell viability or SASP output in some models, whereas in others it alters senescence establishment itself. Autophagy should therefore be considered a potential, model-specific vulnerability rather than a universal dependency.

Alterations in lipid metabolism constitute another important component of the senescent metabolic phenotype. Cells undergoing TIS frequently accumulate intracellular lipid droplets while simultaneously increasing fatty acid uptake and β-oxidation [38,96]. These changes may supply energy, buffer lipotoxic stress, or support membrane and signaling-lipid synthesis, but their functional contribution varies among models. Moreover, lipid-derived signaling molecules produced by senescent cells participate directly in inflammatory signaling, immune modulation, and ECM remodeling, further amplifying the biological effects of the SASP. Recent metabolomic studies have also identified substantial changes in cholesterol metabolism, sphingolipid synthesis, and prostaglandin production, indicating that lipid remodeling represents an integral component of senescence-associated metabolic adaptation.

Nutrient-sensing pathways are central regulators of these metabolic changes. mTOR integrates signals derived from growth factors, nutrient availability, and cellular energy status to coordinate protein synthesis and SASP components. In some senescence models, active mTOR complex 1 (mTORC1) is spatially coupled to autolysosomes, linking metabolite recycling to high secretory output rather than producing a simple opposition between mTOR activity and autophagy. Pharmacological inhibition of mTOR with rapamycin attenuates SASP production without necessarily reversing growth arrest, providing one of the earliest examples of a senomorphic intervention capable of modulating the detrimental consequences of senescence while preserving its tumor-suppressive effects. Conversely, AMP-activated protein kinase (AMPK), which is activated in response to energy depletion, contributes to metabolic adaptation by promoting mitochondrial quality control, regulating autophagy, and limiting excessive anabolic activity. The dynamic balance between mTOR and AMPK signaling therefore determines whether senescent cells maintain metabolic homeostasis or undergo energetic collapse [39,46].

Emerging evidence also implicates nicotinamide adenine dinucleotide (NAD+) metabolism in regulating senescent-cell biology. Senescence can alter NAD+ synthesis and consumption through changes in enzymes including PARPs, sirtuins, CD38, and nicotinamide phosphoribosyltransferase. These changes can affect DNA repair, mitochondrial function, redox homeostasis, and inflammatory signaling. However, both the direction of NAD^+^ change and the consequences of NAD^+^ restoration depend on cell type and senescence trigger. Its contribution to therapy resistance therefore remains unresolved, although the pathway may offer experimentally tractable metabolic targets [70,99,100,101].

Metabolic reprogramming can extend beyond senescent cells and profoundly influence neighboring malignant cells. Metabolites released by senescent cells, together with SASP cytokines and EVs, can alter nutrient use within the TME. In specific experimental systems, senescent or autophagic fibroblasts increase the production of lactate or ketone bodies that can be used by neighboring cancer cells; evidence for a general transfer of glutamine, fatty acids, and other fuels from TIS cells is less established. Such metabolic coupling may support residual tumor-cell survival under nutrient or treatment stress. Senescence-associated metabolic remodeling can therefore operate at both cell-autonomous and tissue levels [102,103].

Collectively, current evidence indicates that metabolic reprogramming can support the establishment and persistence of TIS. Mitochondrial remodeling, autophagy, nutrient sensing, lipid metabolism, and redox homeostasis may sustain senescent-cell survival and modify the microenvironment of residual tumors (Figure 5). These findings identify metabolic pathways as attractive therapeutic targets capable of selectively disrupting senescent-cell survival and potentiating the efficacy of conventional anticancer therapies. These pathways provide candidate vulnerabilities for senolytic or senomorphic intervention, but their selectivity and therapeutic value must be established in appropriate in vivo models and clinical studies [15,38].

### 3.5. Immune Evasion and Failure of Senescence Surveillance Promote Persistence of Therapy-Induced Senescent Cells

The long-term biological consequences of TIS depend not only on the establishment of durable cell-cycle arrest but also on the efficiency with which senescent cells are eliminated by the immune system. Under certain physiological conditions, senescent cells are transient and can be removed through a coordinated process known as senescence surveillance, in which innate and adaptive immune cells recognize and eliminate damaged cells before they accumulate within tissues. This mechanism contributes to maintaining tissue homeostasis and preserving the tumor-suppressive functions of senescence. However, following anticancer therapy, some senescent cells may evade immune clearance, allowing them to persist for prolonged periods and continuously remodel the tumor microenvironment. Increasing evidence suggests that failure of senescence surveillance may contribute to the association of TIS with tumor recurrence and therapeutic resistance [104,105].

The immune recognition of senescent cells is mediated by both the SASP and the cell-surface ligands. During the early stages of senescence, SASP components such as CCL2, CXCL9, CXCL10, granulocyte-macrophage colony-stimulating factor (GM-CSF), IL-15, and type I interferons recruit NK cells, macrophages, dendritic cells, and cytotoxic CD8+ T lymphocytes to sites of tissue damage. Senescent cells also increase the expression of stress-induced ligands, including NKG2D ligands, which enhance recognition by NK cells. Together, these mechanisms establish an immunostimulatory microenvironment that facilitates efficient clearance of damaged cells and contributes to the therapeutic efficacy of chemotherapy and radiotherapy [92,104].

However, this immune-activating phenotype is often transient. As senescent cells persist, profound changes occur in both the composition of the SASP and the immune landscape. Chronic SASP production can shift the TME from an inflammatory state capable of supporting immune surveillance toward one dominated by immune suppression. Persistent secretion of IL-6, IL-8, TGF-β, prostaglandin E2 (PGE2), VEGF, and CCL2 promotes recruitment and expansion of regulatory T cells (Tregs), myeloid-derived suppressor cells (MDSCs), and alternatively activated M2 macrophages. These immunosuppressive populations inhibit cytotoxic T-cell activity, suppress NK-cell function, and create conditions that allow senescent and non-senescent tumor cells to survive despite continued immune infiltration. Consequently, the transition from acute to chronic senescence represents a critical switch in which the same biological program that initially enhances tumor clearance ultimately favors immune escape and therapeutic failure [106,107].

Senescent cells themselves actively participate in immune evasion through alterations in immune checkpoint signaling. Several studies have demonstrated that tumor cells undergoing TIS upregulate immune checkpoint molecules, including programmed death-ligand 1 (PD-L1), which inhibits T-cell activation through engagement of programmed cell death protein 1 (PD-1) receptors on cytotoxic lymphocytes. Additional inhibitory pathways involving HLA-E/NKG2A interactions, CD47-mediated inhibition of phagocytosis, and other immune-regulatory ligands have also been implicated in protecting senescent cells from immune-mediated elimination. These adaptations enable senescent cells to remain viable despite persistent inflammatory signaling and immune cell recruitment, effectively converting an initially immunogenic state into one characterized by immune tolerance and chronic inflammation [108,109].

Macrophages illustrate the dual nature of senescence surveillance particularly well. During effective tissue repair, macrophages efficiently engulf senescent cells and contribute to the resolution of inflammation. In tumors, however, chronic SASP signaling can promote immunosuppressive macrophage states characterized by IL-10 and TGF-β production and ARG1 expression that suppress antitumor immunity while promoting angiogenesis, ECM remodeling, and tumor progression. These tumor-associated macrophages further amplify SASP-mediated inflammation, establishing a self-perpetuating cycle in which senescent cells and immunosuppressive myeloid populations reinforce one another [110,111,112].

NK cells are similarly affected by prolonged exposure to senescent cells. Although NK cells efficiently recognize newly generated senescent cells through activating receptors such as NKG2D, chronic inflammatory signaling progressively impairs NK-cell cytotoxicity, reduces cytokine production, and promotes functional exhaustion. This decline in NK-cell activity is particularly relevant following repeated cycles of chemotherapy or radiotherapy, which can induce systemic lymphopenia and compromise innate immune responses. As a result, residual senescent cells may persist because of impaired immune-mediated elimination over the course of treatment [104,113].

Adaptive immunity is also profoundly influenced by persistent senescence. Chronic antigen exposure sustains inflammatory signaling, and increased expression of immune checkpoint ligands promotes exhaustion of CD8+ T cells, characterized by reduced proliferative capacity, impaired cytokine production, and diminished cytotoxic activity. Simultaneously, expansion of regulatory T cells further suppresses effector T-cell responses, limiting immune-mediated elimination of senescent cells. These changes could influence responsiveness to immune checkpoint blockade, although direct clinical evidence remains limited [108,109].

Another emerging mechanism linking senescence to immune escape involves EVs released by senescent cells. These vesicles transport cytokines, immune-regulatory proteins, microRNAs, and DAMPs capable of modulating immune cell differentiation and function. In experimental models, senescence-associated EVs can impair dendritic cell maturation, inhibit cytotoxic lymphocyte activation, and promote expansion of immunosuppressive myeloid populations, thereby extending the influence of senescent cells beyond their immediate microenvironment. Although this field remains relatively young, EVs-mediated communication is increasingly recognized as an important component of senescence-driven immune remodeling [66,114].

The temporal evolution of senescence surveillance has important therapeutic implications. During the early phases following anticancer treatment, enhancement of immune-mediated clearance may potentiate the beneficial effects of TIS by facilitating complete elimination of damaged tumor cells. In contrast, once chronic senescence has become established, persistent inflammatory signaling and immune suppression may favor tumor recurrence. This concept has led to the proposal that therapeutic interventions should not simply induce senescence but should also promote timely clearance of senescent cells through immune activation or senolytic strategies. Recent combination approaches integrating senescence-inducing therapies with immune checkpoint blockade, adoptive cellular therapies, or senolytic agents have shown encouraging preclinical results, supporting the concept that manipulation of senescence surveillance may substantially improve long-term treatment efficacy [106,115].

Collectively, these findings support immune surveillance as an important, context-dependent regulator of the biological consequences of TIS. Effective clearance of senescent cells preserves the tumor-suppressive functions of senescence, whereas failure of immune surveillance permits persistent SASP production, chronic inflammation, immune suppression, and conditions that may favor tumor relapse. Understanding the molecular mechanisms that govern this transition will be essential for designing therapeutic strategies capable of exploiting senescence without incurring its long-term detrimental consequences [106,115].

## 4. Clinical Evidence Linking Therapy-Induced Senescence to Cancer Therapy Resistance

Although much of the mechanistic understanding of TIS has been derived from experimental models, clinical and translational studies have documented senescence-associated phenotypes after treatment, but direct evidence that these cells cause therapeutic failure in patients remains limited. Analyses of patient biopsies obtained after neoadjuvant chemotherapy, radiotherapy, and targeted therapies have reported increases in senescence-associated biomarkers, including p16INK4a, p21CIP1/WAF1, SA-β-gal, persistent DDR markers such as γH2AX, and transcriptional signatures enriched for inflammatory SASP components. None of these markers is individually specific for senescence. Persistence of these senescence-associated features has been associated in selected cohorts with residual disease, early relapse, metastatic progression, and reduced overall survival, although these associations do not establish TIS as a causal determinant of outcome [84].

One of the principal challenges in translating senescence biology into clinical oncology has been the absence of a single universal biomarker. Senescence is currently identified using combinations of molecular and histological markers, including expression of p16INK4a, p21, loss of proliferative markers such as Ki-67, persistent DDR activation, lipofuscin accumulation, altered lamin B1 expression, and transcriptional profiling of SASP-associated genes. Recent advances in single-cell RNA sequencing, spatial transcriptomics, and multiplex immunohistochemistry have substantially improved the ability to resolve candidate senescent states within heterogeneous human tumors, suggesting that TIS may affect both malignant and stromal compartments. These technologies have further demonstrated considerable heterogeneity among senescent cell populations, indicating that not all senescent cells exert equivalent biological effects [116,117].

### 4.1. Therapy-Induced Senescence Following Chemotherapy

Among conventional anticancer treatments, chemotherapy provides the strongest clinical evidence for TIS. DNA-damaging agents such as doxorubicin, epirubicin, cisplatin, carboplatin, etoposide, irinotecan, temozolomide, and cyclophosphamide can induce extensive DNA damage that activates persistent DDR signaling and senescence in both tumor cells and surrounding stromal cells. Analyses of post-treatment biopsies from patients receiving neoadjuvant chemotherapy have demonstrated increased expression of p21, γH2AX, and SASP-associated inflammatory mediators in residual tumors. These findings document treatment-associated senescence phenotypes but do not establish senescence as the cause of incomplete tumor eradication [84,118].

Breast cancer provides one of the clearest clinical examples of this phenomenon. Following neoadjuvant anthracycline- and taxane-based chemotherapy, residual tumor tissue commonly exhibits increased p21 expression together with inflammatory SASP signatures enriched for IL-6, IL-8, CXCL1, and MMPs. Several studies have associated these signatures with poor pathological response, increased risk of local recurrence, and shorter disease-free survival [119,120]. Similar findings have been reported in other tumor types, particularly ovarian cancer, where experimental studies have associated chemotherapy-induced senescence of stromal fibroblasts with a pro-tumorigenic microenvironment and enhanced invasive and clonogenic properties [119].

### 4.2. Radiotherapy-Induced Senescence

Ionizing radiation is another potent inducer of cellular senescence through the generation of persistent DNA double-strand breaks and oxidative stress. While radiation-induced senescence contributes substantially to local tumor control, it also promotes long-lasting remodeling of irradiated tissues. Experimental and translational studies indicate that radiotherapy can induce senescence in fibroblasts and other stromal and immune-cell populations [121,122].

Persistent radiation-induced SASP has been implicated in fibrosis, chronic inflammation, vascular remodeling, and the establishment of a microenvironment that favors the survival of residual malignant cells [123,124]. Elevated inflammatory and fibrotic mediators, including IL-6 and TGF-β, have been implicated in radiation-induced tissue injury and fibrosis, while experimental studies indicate that persistent SASP signaling can promote a microenvironment conducive to tumor-cell survival, radioresistance, and recurrence [122]. These observations suggest that the long-term consequences of radiotherapy extend beyond direct tumor cell killing and involve sustained alterations in the surrounding tissue environment [124].

### 4.3. Targeted Therapies and Senescence

Several targeted therapies also induce senescence independently of direct DNA damage. CDK4/6 inhibitors represent perhaps the best-characterized example. Agents such as palbociclib, ribociclib, and abemaciclib can induce durable proliferative arrest and senescence-associated features, although a robust SASP is not universal. The SASP is substantially more heterogeneous than the durable cell-cycle arrest, and different CDK4/6 inhibitors and cellular contexts can produce different secretory phenotypes. This antiproliferative effect contributes to prolonged disease stabilization in hormone receptor-positive breast cancer, whereas persistent senescent cells may eventually acquire inflammatory phenotypes that facilitate adaptive resistance. Similar mechanisms have been described in preclinical models following inhibition of BRAF/MEK and EGFR, where treatment-induced senescence is accompanied by secretion of inflammatory mediators capable of promoting survival of resistant tumor clones [28,125,126,127].

These observations indicate that resistance to targeted therapies does not arise exclusively through secondary genetic mutations. Reversible, non-genetic mechanisms, including cellular plasticity, adaptive drug-tolerant states, inflammatory signaling, and microenvironmental remodeling, can contribute to treatment failure. TIS represents one potential non-genetic state through which residual tumor and stromal cells can influence subsequent disease behavior. This distinction has important therapeutic implications because non-genetic resistance states may be more reversible than fixed genetic resistance, raising the possibility that senescent-cell elimination or SASP modulation could improve the durability of targeted therapies [84,128].

### 4.4. Therapy-Induced Senescence and Immunotherapy

The relationship between senescence and immunotherapy remains more complex and is only beginning to be understood. On one hand, senescence enhances antigen presentation and promotes recruitment of immune effector cells through inflammatory cytokine production, potentially increasing tumor immunogenicity. On the other hand, persistent senescence favors immune suppression through expansion of Tregs, MDSCs, and M2 macrophages and can simultaneously increase expression of immune-regulatory molecules such as PD-L1 in some contexts [85]. Consequently, TIS could exert opposing effects on immune checkpoint blockade; whether these effects change over time remains unresolved [129,130].

Emerging studies have identified associations between senescence-associated transcriptional programs and immune checkpoint response across several tumor types, including melanoma, lung cancer, renal cell carcinoma, and hepatocellular carcinoma. However, whether these signatures directly reflect persistent senescent cells or broader features of tumor inflammation and immune suppression remains an important unresolved question [55,131].

### 4.5. Senescence Biomarkers as Predictors of Clinical Outcome

Beyond their biological significance, senescence-associated biomarkers are increasingly being explored as prognostic and predictive biomarkers. High intratumoral expression of p16INK4a, persistent p21 induction, elevated circulating IL-6 and IL-8 concentrations, and transcriptional enrichment of SASP-related genes have been associated with clinical outcome in selected malignancies and patient populations. Nevertheless, interpretation of these biomarkers remains challenging because none is specific for senescence, and their prognostic meaning is highly context-dependent. Development of standardized senescence signatures integrating molecular, histological, and functional parameters will therefore be essential before senescence can be routinely incorporated into clinical decision-making [84,132].

Collectively, available clinical and translational evidence supports the relevance of TIS in human cancer, while also emphasizing that its effects are highly context-dependent (Table 2). Although induction of senescence can contribute to the immediate efficacy of anticancer treatments through durable proliferative arrest, persistence of senescent cells and their SASP may subsequently promote tumor-cell survival, inflammatory remodeling, therapeutic resistance, and, in some settings, recurrence. These findings provide a rationale for therapeutic strategies designed to selectively eliminate persistent senescent cells or suppress deleterious SASP signaling following conventional anticancer therapy. However, senolytic and senomorphic strategies remain largely preclinical, and their optimal timing, specificity, and safety in patients require further investigation [133,134].

To provide a more clinically informative overview of the available evidence, we have included Table S1 (Supplementary Information: Clinical and translational evidence linking cancer-associated senescence with treatment response, resistance, and clinical outcomes). Table 2 has been maintained as a summary of the methodological and clinical characteristics of studies investigating cancer-associated senescence in the context of treatment response, treatment resistance, and patient outcomes. In Table S1, in addition to cancer type, treatment modality, and senescence-associated markers, the table now reports, where available, the number of patients or participants, study design, biological sample analyzed, timing of sample collection, cellular localization of the senescence-associated signal (tumor and/or stromal cells), clinical endpoint or outcome assessed, and whether the findings were independently validated. These parameters are included to facilitate distinction between exploratory observations, translational evidence, and clinically validated associations.

## 5. Senolytic Therapies: Selective Elimination of Persistent Senescent Cells

The observation that senescent cells depend on specific pro-survival signaling pathways to resist apoptosis has provided the biological rationale for the development of senolytic agents. Unlike conventional cytotoxic drugs, which preferentially target rapidly proliferating cells, senolytics selectively eliminate senescent cells by disrupting the adaptive mechanisms that enable their long-term survival. These compounds exploit the increased dependence of senescent cells on anti-apoptotic pathways, collectively referred to as senescent cell anti-apoptotic pathways (SCAPs), thereby inducing apoptosis preferentially in susceptible senescent cells; their selectivity depends on cell type and senescence context [49,135].

Senescent cells survive despite extensive DNA damage, mitochondrial dysfunction, oxidative stress, and chronic inflammatory signaling because they activate multiple anti-apoptotic networks. Among the best characterized are members of the BCL-2 protein family (BCL-2, BCL-xL, and BCL-W), the PI3K/AKT pathway, HSP90-mediated protein stabilization, ephrin signaling, and regulators of mitochondrial integrity. Although these pathways differ among cell types and senescence-inducing stimuli, their coordinated activation creates a state of apoptotic resistance that represents a defining characteristic of persistent senescent cells (Figure 6). Consequently, pharmacological inhibition of SCAPs can preferentially sensitize senescent cells to apoptosis, although the resulting therapeutic window is context-dependent [49].

One of the first senolytic compounds to demonstrate robust activity was navitoclax (ABT-263), a small-molecule inhibitor of BCL-2, BCL-xL, and BCL-W. Navitoclax effectively eliminates tumor cells undergoing TIS in numerous preclinical models, including breast, lung, hematological, and colorectal malignancies. Experimental studies have shown that sequential administration of navitoclax following chemotherapy or radiotherapy reduces the burden of persistent senescent cells, attenuates SASP production, delays tumor recurrence, and improves long-term treatment responses [49]. These findings have established BCL-2 family proteins as central therapeutic targets in senolytic strategies.

Despite these encouraging results, clinical translation of navitoclax has been complicated by dose-limiting thrombocytopenia resulting from the dependence of platelets on BCL-xL for survival. This toxicity has stimulated the development of more selective BCL-xL inhibitors, proteolysis-targeting chimeras (PROTACs), and antibody-directed delivery systems designed to preserve senolytic efficacy while minimizing hematological adverse effects [49]. These next-generation compounds remain under active preclinical and early translational investigation [136].

Another widely studied senolytic approach combines dasatinib, a broad-spectrum tyrosine kinase inhibitor, with the flavonoid quercetin. Originally identified through transcriptomic analyses of senescent cells, this combination targets complementary SCAP networks and has demonstrated senolytic activity across multiple experimental models [137,138]. Dasatinib appears particularly effective against senescent adipocyte progenitors and certain tumor cells, whereas quercetin preferentially targets senescent endothelial cells and fibroblasts through inhibition of PI3K, serpines, and other survival pathways. Together, these compounds reduce senescent-cell burden, suppress systemic inflammation, and improve tissue function in several models of aging and therapy-induced toxicity. Clinical evidence is currently limited to small early-phase studies in non-oncological conditions; these studies support feasibility and short-term tolerability but do not establish antitumor efficacy or a broadly favorable safety profile [137,138,139].

Natural compounds with putative senolytic activity have also attracted considerable interest. Among these, sisetin, a naturally occurring flavonoid present in fruits and vegetables, has demonstrated senolytic activity in selected experimental models while reducing systemic inflammation and improving physical function in aged animals. Additional compounds, including piperlongumine, cardiac glycosides, bromodomain and extraterminal (BET) protein inhibitors, and HSP90 inhibitors, have shown varying degrees of senolytic activity depending on cell type and senescence context. However, most remain at the preclinical stage, and their selectivity toward tumour cells undergoing TIS requires further investigation [49,137].

Recent advances have expanded senolytic strategies beyond small molecules. One particularly innovative approach involves engineering chimeric antigen receptor (CAR) T cells capable of recognizing surface proteins enriched in selected senescent-cell populations. Amor and colleagues [140] demonstrated that urokinase-type plasminogen activator receptor (uPAR)-directed CAR T cells targeted senescent cells in mouse models of liver fibrosis and TIS in lung adenocarcinoma, reducing fibrosis and extending survival, respectively. Thus, the original proof-of-concept study included both non-malignant and oncological models; however, target specificity and safety require further validation before clinical translation [141]. Similar approaches employing antibody-drug conjugates, bispecific antibodies, and NK-cell-based therapies are currently under investigation.

Despite remarkable progress, several challenges continue to limit the clinical application of senolytics. Senescent cells are highly heterogeneous, and their dependence on individual SCAPs varies according to tumor type, tissue origin, and senescence-inducing stimulus. Consequently, no single senolytic agent is likely to eliminate all senescent cell populations. Furthermore, transient senescence contributes to tissue repair and wound healing; indiscriminate elimination of newly generated senescent cells could therefore interfere with physiological regeneration or compromise beneficial antitumor immune responses. These observations suggest that successful senolytic therapy will require careful optimization of treatment timing, patient selection, and biomarker-guided identification of persistent pathological senescent cells [49,137,140].

Nevertheless, accumulating preclinical evidence supports the therapeutic potential of senolytics in oncology. By selectively eliminating the persistent senescent cells that remain after chemotherapy, radiotherapy, or targeted therapy, senolytic agents may have the potential to interrupt the cascade linking TIS to chronic SASP production, CSC expansion, immune suppression, and eventual tumor relapse. Rather than replacing conventional anticancer therapies, senolytics are therefore viewed as complementary interventions designed to consolidate initial treatment responses by removing the residual senescent cell populations that may sustain long-term therapeutic resistance [49,137,140].

## 6. Senomorphic Therapies: Modulating the Senescent Phenotype Without Eliminating Senescent Cells

Although senolytic therapies aim to eradicate persistent senescent cells, complete elimination of senescent cells may not always be desirable. Acute senescence plays essential physiological roles in tissue repair, wound healing, embryonic development, and tumor suppression [137]. Consequently, indiscriminate removal of all senescent cells could compromise beneficial biological processes, particularly when senescent cells contribute to transient physiological repair responses. This concern has stimulated the development of senomorphic therapies, which suppress the deleterious features of senescence, particularly the SASP, while preserving stable cell-cycle arrest [105].

Unlike senolytics, senomorphic agents do not directly induce apoptosis of senescent cells. Instead, they target the signaling pathways responsible for maintaining the inflammatory phenotype and metabolic activity characteristic of senescence. By attenuating SASP production, senomorphics seek to preserve the tumor-suppressive effects of TIS while potentially limiting microenvironmental conditions that support tumor persistence and therapeutic resistance [105,137].

One of the best-characterized regulators of the SASP is the mTOR pathway. Beyond its well-established role in controlling cellular metabolism and protein synthesis, mTOR regulates the translation of numerous inflammatory mediators secreted by senescent cells. Pharmacological inhibition of mTOR with rapamycin or its analogs significantly reduces production of IL-6, IL-8, VEGF, and MMPs without reversing growth arrest. These observations demonstrate that proliferative arrest and inflammatory signaling can be pharmacologically dissociated; however, preservation of growth arrest does not by itself establish that tumor-suppressive function is maintained in vivo [39,46]. In additional preclinical models, mTOR inhibition has also been reported to attenuate aspects of stromal activation and radiation-associated tissue remodeling, supporting the possibility that senomorphic approaches could have applications beyond direct modulation of tumor-cell senescence.

Another important target is the NF-κB signaling pathway, widely recognized as a major transcriptional regulator of the SASP. Persistent DDR signaling, mitochondrial dysfunction, and activation of the cGAS-STING pathway converge on NF-κB, driving sustained transcription of pro-inflammatory cytokines, chemokines, and proteases. Inhibition of NF-κB signaling reduces expression of IL-1α, IL-6, IL-8, CXCL1, CCL2, and numerous matrix-remodeling enzymes, thereby attenuating the ability of senescent cells to remodel the TME [142,143]. Although systemic inhibition of NF-κB remains challenging because of its central role in innate immunity, selective modulation of this pathway continues to be actively investigated as a strategy for limiting chronic SASP production.

The JAK/STAT pathway represents another attractive therapeutic target. Several major SASP cytokines, particularly IL-6, signal through JAK/STAT3, establishing positive feedback loops that reinforce inflammatory signaling and promote cancer stemness. Pharmacological inhibition of JAK1/2 suppresses SASP production, reduces inflammatory signaling, and improves tissue function in experimental models of senescence [144,145]. These findings have generated interest in clinically approved JAK inhibitors, including ruxolitinib, as potential senomorphic agents, although their efficacy against TIS in cancer remains to be established.

The p38 MAPK pathway also contributes significantly to SASP regulation. Activation of p38 MAPK occurs downstream of persistent DNA damage, oxidative stress, and mitochondrial dysfunction, promoting stabilization of inflammatory transcripts and activation of NF-κB. Pharmacological inhibition of p38 reduces secretion of pro-inflammatory cytokines and attenuates senescence-associated inflammation in several preclinical models [143]. Thus, p38 inhibition remains an important experimental strategy for dissecting the molecular regulation of the SASP.

Increasing attention has also focused on metformin, a widely prescribed antidiabetic drug that exhibits multiple senomorphic properties. Metformin can modulate AMPK–mTOR signaling and mitochondrial metabolism; its experimentally demonstrated senomorphic activity includes suppression of selected SASP components through interference with IKK–NF-κB signaling. Several epidemiological studies have suggested that metformin use is associated with improved cancer outcomes and reduced incidence of age-related diseases, although the precise contribution of senescence modulation to these effects remains under investigation. Because of its extensive clinical experience, relatively well-characterized safety profile, and effects on multiple pathways implicated in senescence, metformin represents an attractive candidate for investigation in combination strategies aimed at controlling chronic senescence-associated inflammation [146,147,148].

Beyond classical signaling inhibitors, modulation of the cGAS-STING pathway has emerged as an area of considerable interest. Cytoplasmic chromatin fragments generated during TIS activate cGAS, leading to production of cyclic GMP–STING activation can promote immune surveillance of senescent cells; persistent activation contributes to chronic inflammation and sustained SASP production. The dual role of this pathway highlights the complexity of senomorphic therapy, as excessive inhibition may compromise beneficial antitumor immunity, whereas insufficient inhibition may permit chronic inflammatory remodeling [47,149]. These findings suggest that the timing, magnitude, and context of cGAS–STING modulation are likely to be important determinants of therapeutic efficacy.

An additional challenge for senomorphic therapy is the remarkable heterogeneity of the SASP itself. The composition of the secretome varies according to tumor type, genetic background, therapeutic modality, and duration of senescence [50,105]. Consequently, inhibition of a single signaling pathway is unlikely to completely suppress the diverse biological activities of the SASP. Multi-target approaches simultaneously modulating mTOR, NF-κB, JAK/STAT, and metabolic signaling may therefore prove more effective than single-agent therapies. Advances in transcriptomic and proteomic profiling of senescent cells are expected to facilitate identification of context-specific SASP signatures that can guide personalized senomorphic interventions.

Despite their considerable promise, senomorphics also present important limitations. Because senescent cells remain viable, continued accumulation of these cells may eventually allow recovery of inflammatory signaling or facilitate senescence escape once treatment is discontinued. Furthermore, prolonged suppression of inflammatory pathways could reduce immune-mediated clearance of senescent cells, potentially prolonging their persistence within tissues. These observations suggest that senomorphic therapies may be most effective when administered transiently during periods of maximal SASP activity or when combined with strategies that subsequently eliminate senescent cells [7,49,137,150].

Collectively, senomorphic agents represent a complementary therapeutic strategy that seeks to attenuate the pathological consequences of TIS while preserving stable growth arrest; whether the antitumor effects of senescence are also preserved must be established in each context (Table 3). By selectively suppressing inflammatory signaling, stromal remodeling, and microenvironmental adaptation, these compounds may reduce the development of therapy resistance, although a safety advantage over senolytic strategies has not yet been demonstrated. Future clinical studies will determine whether senomorphics are best employed as standalone interventions or as components of sequential combination strategies integrating conventional therapy, immune modulation, and senolytic treatment [49,105].

## 7. The “One–Two Punch” Strategy: Sequential Targeting of Therapy-Induced Senescence

The growing recognition that persistent TIS cells can contribute in preclinical models to tumor recurrence has led to the development of an innovative therapeutic paradigm known as the “one–two punch” strategy. Rather than considering senescence as the final objective of cancer treatment, this approach deliberately exploits its dual biological nature. In the first step (“first punch”), conventional anticancer therapies induce senescence in tumor cells, thereby halting proliferation and limiting tumor growth. In the second step (“second punch”), senescent cells are selectively eliminated using senolytic agents or other senescence-targeting interventions before they can establish a chronic pro-tumorigenic microenvironment [49,137,151].

Evidence status. The “one–two punch” should currently be regarded as a translational framework rather than an established clinical treatment strategy. The strongest evidence comes from experimental and animal models in which senescence induction is followed by senescent-cell elimination or functional modulation. Early human studies of individual senotherapeutic agents provide information about feasibility, safety, or biological activity, but they do not establish that sequential senescence targeting improves cancer survival. Accordingly, claims of therapeutic benefit are restricted to the level supported by the underlying evidence.

This strategy is based on the premise that senescence is initially beneficial but becomes detrimental if senescent cells persist. During the acute phase, TIS contributes to tumor suppression by inducing stable cell-cycle arrest, promoting immune-mediated clearance of damaged cells, and preventing immediate expansion of genetically unstable clones. However, prolonged persistence of senescent cells results in sustained SASP production, chronic inflammation, induction of EMT, acquisition of stem cell-like phenotypes, immune suppression, and, in some cases, escape from senescence with re-entry into the cell cycle. Consequently, therapeutic success may depend not only on inducing senescence but also on ensuring the timely removal of senescent cells before these detrimental processes become established [49,137,151].

Preclinical studies have provided compelling evidence supporting this sequential approach. In murine models of breast cancer, lymphoma, and lung cancer, administration of the BCL-2/BCL-xL inhibitor navitoclax (ABT-263) following chemotherapy markedly reduced the burden of senescent cells, attenuated SASP production, and delayed tumor recurrence compared with chemotherapy alone [152,153]. Similarly, sequential treatment with dasatinib plus quercetin (D+Q) after senescence-inducing therapies has reduced senescent cell accumulation, improved tissue homeostasis, and limited therapy-induced toxicities in several experimental models, although its direct antitumor activity appears to be more context-dependent [49,154].

The efficacy of the “one–two punch” strategy has also been demonstrated in targeted therapy models. In breast cancer, CDK4/6 inhibitors can induce durable proliferative arrest and senescence-associated features, although the extent and composition of the SASP are context-dependent. While the antiproliferative effect of CDK4/6 inhibition contributes to prolonged disease stabilization, persistent senescent cells may subsequently promote inflammatory remodeling and adaptive resistance. Preclinical studies have shown that administration of senolytic agents after CDK4/6 inhibition selectively eliminates senescent tumor cells, decreases inflammatory signaling, and enhances durable tumor control [131,155]. Similar observations have been reported in preclinical models following treatment with PARP inhibitors, BRAF inhibitors, and EGFR-targeted therapies, suggesting that sequential senescence targeting may have broad applicability across therapeutic modalities.

Radiotherapy represents another setting in which the “one–two punch” concept may be particularly advantageous. Ionizing radiation efficiently induces senescence in both tumor and stromal cells, but persistent senescent fibroblasts contribute to radiation-induced fibrosis, chronic inflammation, and remodeling of the TME. Experimental studies have demonstrated that senolytic administration after irradiation reduces tissue fibrosis, improves normal tissue function, and decreases recurrence in preclinical models, raising the possibility that senescence-targeting strategies may simultaneously enhance antitumor efficacy while reducing late treatment-related toxicities [43,156].

An essential aspect of this therapeutic paradigm is treatment timing. Premature elimination of senescent cells could interfere with the beneficial tumor-suppressive effects of acute senescence, including SASP-dependent immune recruitment and tissue repair. Conversely, excessive delays may allow chronic SASP signaling to promote cellular plasticity, cancer stemness, immune evasion, and senescence escape. Defining the optimal therapeutic window therefore represents one of the major translational challenges in the field. Biomarkers capable of distinguishing transient, beneficial senescence from persistent pathological senescence are likely to be critical for determining when senolytic treatment should be initiated.

Recent studies suggest that dynamic biomarker monitoring, rather than single time-point measurements, may provide the most informative approach. Longitudinal assessment of circulating SASP factors (e.g., IL-6, IL-8, CCL2), senescence-associated EVs, circulating tumor DNA as a complementary measure of tumor burden, and molecular imaging of senescent cells could potentially help guide senolytic intervention in individual patients. Advances in single-cell transcriptomics and spatial multi-omics are also beginning to identify senescence-associated molecular signatures that distinguish heterogeneous senescent populations with distinct biological functions, opening the possibility of precision senescence-targeted therapy [50,132]. However, none of these approaches has yet been clinically validated to determine the timing of senolytic treatment.

The concept of the “one–two punch” has recently expanded beyond pharmacological senolysis. An emerging strategy combines senescence induction with immune-mediated clearance, effectively creating an “immune second punch.” Senescence can alter antigen presentation and inflammatory signaling and, in selected contexts, enhance immune recognition. Subsequent administration of immune checkpoint inhibitors, NK cell therapies, macrophage-directed approaches, or CAR T cells targeting senescence-enriched surface antigens may then augment physiological senescence surveillance, facilitating selective elimination of persistent senescent cells [140,157]. This integrated strategy may overcome some of the limitations associated with small-molecule senolytics, particularly in heterogeneous tumors containing multiple senescent cell populations.

Despite its considerable promise, the “one–two punch” approach faces several important challenges before widespread clinical implementation [49,132,137]. First, senescent cells exhibit substantial heterogeneity, and their dependence on SCAPs varies according to tumor type, genetic background, and treatment modality. Second, currently available senolytics may also affect non-senescent cells, leading to adverse effects such as thrombocytopenia observed with navitoclax. Third, reliable and clinically validated biomarkers capable of identifying persistent pathological senescence remain limited. Finally, prospective clinical trials specifically designed to evaluate sequential senescence-targeting strategies are scarce.

Nevertheless, the “one–two punch” paradigm represents a compelling translational framework for applying senescence biology in oncology (Figure 7). By combining conventional cancer therapy with subsequent elimination or functional modulation of persistent senescent cells, this strategy seeks to preserve the initial tumor-suppressive effects of senescence while limiting its potential contribution to long-term therapeutic resistance. Rather than viewing senescence as intrinsically beneficial or detrimental, the “one–two punch” model emphasizes that its therapeutic consequences depend on the cellular context, duration, and fate of the senescent state [84,85,137]. Successful application of this concept will therefore require not only effective induction of senescence but also accurate identification and controlled management of persistent senescent populations.

## 8. Clinical Translation of Senescence-Targeted Therapies: Current Progress and Ongoing Challenges

The remarkable advances in understanding the biology of TIS have stimulated considerable interest in translating senescence-targeted therapies into clinical oncology. Although preclinical studies consistently suggest that eliminating or modulating persistent senescent cells can improve therapeutic responses and reduce tumor recurrence, clinical implementation remains at an early stage. Recent critical assessments emphasize that, despite substantial preclinical evidence supporting senolytic strategies, their clinical utility in cancer has not yet been established. This field is therefore transitioning from proof-of-concept studies toward carefully designed clinical trials addressing efficacy, safety, treatment timing, and patient selection. The biological consequences of TIS are context-dependent: senescence can suppress tumor growth through durable cell-cycle arrest, while persistent senescent cells and their secretory phenotype may promote inflammation, immune dysfunction, and tumor progression [3,158].

One of the major barriers to clinical translation is the absence of validated biomarkers capable of reliably identifying senescent cells in human tumors. Unlike apoptosis or proliferation, senescence cannot be defined by a single molecular marker. Although expression of p16INK4a, p21CIP1/WAF1, SA-β-gal, γH2AX, loss of Lamin B1, accumulation of lipofuscin, and SASP-associated cytokines are widely used experimentally, each marker has important limitations regarding specificity, sensitivity, and tissue applicability. Consequently, current consensus emphasizes the use of combinations of markers reflecting complementary aspects of the senescent phenotype rather than reliance on a single biomarker [3,116,159]. Furthermore, senescence is highly heterogeneous, and distinct senescent populations can coexist within the same tumor. The development of standardized biomarker panels integrating histological, transcriptomic, proteomic, and metabolomic signatures therefore represents a major priority for clinical implementation.

Recent advances in single-cell RNA sequencing, spatial transcriptomics, mass cytometry, and multiplex imaging have substantially improved the resolution and characterization of candidate senescent states within complex tissues. These approaches indicate that senescence is not restricted to malignant cells but can involve fibroblasts, endothelial cells, immune cells, and other stromal populations, each potentially displaying distinct senescence-associated transcriptional and secretory programs. For example, integrated single-cell and spatial transcriptomic analyses of hepatocellular carcinoma have demonstrated substantial heterogeneity in senescence-associated programs across cell types within the TME, with particularly prominent features in endothelial cells [160]. Similarly, computational integration of single-cell and spatial transcriptomic data has enabled improved spatial mapping of cells with senescence-associated signatures and their cellular interactions [161]. These findings support the concept that future senescence-targeted therapies will likely require personalized approaches adapted to the cellular composition and molecular characteristics of individual tumors rather than universal treatment strategies.

Another critical issue concerns the optimal timing of intervention. The therapeutic value of senescence is highly dependent on its temporal dynamics. Acute TIS may contribute to tumor suppression through stable growth arrest and subsequent immune-mediated clearance, whereas chronic persistence can promote inflammatory signaling, immune suppression, EMT, and therapy resistance [84,158,162]. Experimental studies have further demonstrated that senescent tumor cells can escape growth arrest and contribute to tumor recurrence, supporting the rationale for sequential senolytic treatment after senescence-inducing therapy [162,163]. Consequently, senolytic therapy administered too early could theoretically compromise beneficial antitumor effects associated with TIS, whereas excessively delayed intervention may permit persistent senescent cells to remodel the TME. Determining the appropriate therapeutic window therefore represents one of the most important unanswered questions in the field.

Clinical trial design presents additional challenges. Much of the human evidence for senolytic therapy has emerged from age-related disorders and chronic diseases rather than oncology. For example, a phase I pilot study of dasatinib plus quercetin (D+Q) in patients with diabetic kidney disease demonstrated reductions in several measures of senescent-cell burden, providing early evidence that pharmacological senolysis can be achieved in humans [139]. However, these findings cannot be directly extrapolated to cancer patients, who present unique biological complexities including extensive intratumoral heterogeneity, treatment-induced immunosuppression, organ-specific toxicity, and repeated exposure to cytotoxic or targeted therapies. Thus, clinical evidence from aging and chronic disease provides important information concerning feasibility and safety but does not establish the efficacy of senescence-targeted approaches in oncology.

Several compounds currently under clinical investigation illustrate both the promise and limitations of senescence-targeted therapies. Navitoclax (ABT-263) was initially developed as a BCL-2 family inhibitor and subsequently identified as a senolytic agent capable of preferentially eliminating senescent cells [154]. In oncology, navitoclax has already undergone phase I evaluation in patients with solid tumors and hematological malignancies, but dose-dependent thrombocytopenia has represented a major limitation because of BCL-xL dependence in platelets [164,165]. These early oncology trials preceded the recognition of navitoclax as a senolytic and therefore should not be interpreted as clinical validation of senolytic therapy per se. Nevertheless, the existing clinical pharmacology of navitoclax provides an important foundation for evaluating its potential use in senescence-targeted combinations. This toxicity has stimulated the development of more selective BCL-xL degraders and senolytic prodrugs. For example, PROTAC-based approaches have been shown to reduce platelet toxicity while retaining or enhancing senolytic activity in preclinical models [166,167], while galacto-conjugated navitoclax has been designed to exploit elevated lysosomal β-galactosidase activity in senescent cells and thereby improve selectivity [168].

Similarly, D+Q has demonstrated biological activity and acceptable short-term tolerability in several early clinical studies outside oncology, including studies of pulmonary fibrosis and diabetic kidney disease [139]. However, its ability to eliminate tumor cells undergoing TIS in patients and translate into improved oncological outcomes remains uncertain. Other candidate senolytics, including fisetin, HSP90 inhibitors, cardiac glycosides, and agents targeting FOXO4–p53 interactions, remain predominantly in preclinical or early translational development. Fisetin, for example, has demonstrated senotherapeutic activity in experimental models and ex vivo human adipose-tissue explants but has not yet established clinical efficacy as an anticancer senolytic [169]. HSP90 inhibitors were identified as a distinct class of senolytic agents in preclinical studies [170], whereas disruption of the FOXO4–p53 interaction has provided another mechanistically distinct approach to senescent-cell elimination [171]. These findings highlight the diversity of potential senolytic mechanisms but also emphasize the substantial gap between experimental efficacy and clinical validation.

Beyond pharmacological approaches, immune-based therapies targeting senescent cells represent one of the fastest-growing areas of investigation. CAR T cells directed against uPAR have demonstrated the ability to recognize and eliminate senescent cells in experimental models, including models of cancer and fibrosis [140]. In particular, uPAR-targeted CAR T cells improved survival in mice bearing lung adenocarcinoma treated with senescence-inducing therapy, providing proof-of-concept that immune-mediated senolysis could complement conventional anticancer treatment [140]. Subsequent studies have extended this concept to aging-associated metabolic dysfunction and other pathological contexts [172]. Antibody-based strategies directed against senescence-associated surface proteins and approaches designed to enhance NK-cell-mediated surveillance are also being investigated. These approaches offer the possibility of greater targeting precision than conventional small-molecule senolytics while simultaneously engaging endogenous antitumor immunity. However, uPAR is enriched in selected senescent populations rather than being specific to senescence. Nevertheless, antigen heterogeneity, on-target/off-tumor effects, immune-related toxicity, persistence, and the potential for antigen escape remain important barriers to clinical application.

The increasing integration of artificial intelligence (AI) and computational biology into oncology may further accelerate clinical translation. Machine-learning algorithms applied to digital pathology, transcriptomic datasets, and multi-omics analyses are beginning to identify senescence-associated molecular signatures with greater resolution than conventional histopathological evaluation. Recent single-cell and spatial transcriptomic studies demonstrate the feasibility of computationally identifying senescence-associated cellular states and their spatial organization within tumors [160,161]. Such approaches may ultimately facilitate patient stratification, predict responsiveness to senescence-targeted therapies, and optimize treatment scheduling. Nevertheless, computational signatures require rigorous external validation, standardized definitions of senescence, and prospective correlation with clinical outcomes before they can be incorporated reliably into precision oncology.

Another important consideration is that senescence should no longer be viewed as an isolated biological process but rather as one component of the broader adaptive response of tumors to therapy. TIS interacts extensively with DDR, metabolic reprogramming, EMT, immune checkpoint signaling, and CSC biology. Persistent senescent cells can also influence immune-cell composition and function within the TME, potentially contributing to resistance to immunotherapy [162,173]. Consequently, senescence-targeted therapies are unlikely to achieve maximal efficacy as monotherapies. Instead, future treatment strategies will probably integrate senolytics or senomorphics with chemotherapy, radiotherapy, targeted therapy, immunotherapy, and metabolic interventions in rationally designed sequential or combination regimens. Preclinical studies already support sequential approaches in which a senescence-inducing treatment is followed by senolytic clearance of residual senescent tumor cells [163].

Despite the considerable enthusiasm surrounding senotherapeutics, several unresolved questions continue to limit clinical implementation. First, the molecular heterogeneity of senescent cells remains incompletely understood, and senescence-associated phenotypes may vary substantially according to cell type, inducing stimulus, tissue context, and duration. Second, the mechanisms responsible for senescence escape require further clarification because persistent senescent tumor cells may regain proliferative capacity and contribute to recurrence [162]. Third, reliable methods for monitoring senescent-cell burden during treatment are still lacking, underscoring the need for validated tissue and circulating biomarkers [116]. Finally, prospective randomized clinical trials specifically designed to evaluate whether senescence-targeted interventions improve clinically meaningful endpoints such as progression-free survival, recurrence-free survival, or overall survival remain limited. These gaps are particularly important because the current evidence base is dominated by preclinical studies and small early-phase trials, making it difficult to determine the optimal patient population, treatment sequence, and therapeutic window.

Collectively, current evidence indicates that senescence-targeted therapies represent a promising translational strategy emerging from contemporary cancer biology. However, the field has not yet demonstrated that senolysis or senomorphic modulation improves survival outcomes in patients with cancer. Successful clinical implementation will require validated multidimensional biomarkers, improved understanding of senescence heterogeneity, optimized treatment scheduling, more selective therapeutic agents, and carefully designed clinical trials capable of identifying patients most likely to benefit from modulation of TIS. As emphasized by recent critical analyses, the key translational challenge is no longer simply demonstrating that senescent cells can be eliminated, but determining which senescent cells should be targeted, when they should be targeted, and whether their elimination produces a clinically meaningful therapeutic benefit [162].

Limitations of the evidence base. The literature reviewed here is characterized by substantial biological and methodological heterogeneity. Senescence definitions vary across models, tissues, and studies; many biomarkers are not specific; and clinical cohorts are often small or observational (Table 4). Consequently, a senescence-associated phenotype should not automatically be interpreted as the cause of treatment resistance, and promising preclinical senolytic or senomorphic effects should not be equated with demonstrated clinical benefit. These limitations motivate the need for standardized multimodal biomarkers and prospective, biomarker-guided clinical trials.

Table 4 Selected Senescence-Targeted Therapeutic Strategies in Clinical and Preclinical Development.

## 9. Questions and Future Directions

Over the past two decades, TIS has evolved from being viewed primarily as a desirable endpoint of anticancer therapy to being recognized as a dynamic biological state with both tumor-suppressive and potentially tumor-promoting consequences. This conceptual shift has profoundly influenced our understanding of cancer therapy resistance and has stimulated the development of senescence-targeted therapeutic strategies. Senescence can restrict tumor growth through durable cell-cycle arrest, while persistent senescent cells can modify the TME through their secretory phenotype and potentially contribute to tumor progression, recurrence, and metastasis [3,174]. Nevertheless, despite substantial progress in elucidating the molecular mechanisms underlying TIS, several fundamental questions remain unresolved. Addressing these challenges will be essential for translating senescence biology into effective clinical interventions.

### 9.1. Defining the Beneficial vs. Detrimental Phases of Therapy-Induced Senescence

One of the central unresolved questions concerns the temporal nature of TIS. Increasing evidence indicates that senescence can contribute to tumor suppression by establishing durable cell-cycle arrest and facilitating immune-mediated clearance, while the prolonged persistence of senescent cells can promote chronic inflammatory signaling, ECM remodeling, immune dysfunction, and tumor-supportive microenvironmental changes [3,158,174]. The biological consequences of senescence are therefore likely to depend not only on the induction of the state but also on its duration, cellular context, and efficiency of immune clearance.

Despite this conceptual framework, the precise transition from predominantly beneficial to potentially detrimental senescence remains poorly defined. It is currently unknown whether this transition occurs after a reproducible temporal interval or instead varies according to tumor type, therapeutic modality, genetic background, immune competence, and characteristics of the TME. Moreover, senescence is not necessarily an irreversible and static endpoint; under certain experimental conditions, senescent tumor cells can acquire proliferative capacity and contribute to tumor relapse, emphasizing the importance of understanding senescence stability and escape [162]. Understanding the kinetics of senescence establishment, persistence, immune clearance, and escape will therefore be critical for determining the optimal timing of senescence-targeted interventions.

Future studies should integrate longitudinal sampling, single-cell analyses, spatial profiling, and computational modeling to characterize the temporal evolution of senescent cell populations in patients undergoing anticancer therapy. Such approaches may help distinguish transient senescence associated with therapeutic benefit from persistent senescence associated with adverse remodeling of the TME.

### 9.2. The Need for Reliable and Standardized Senescence Biomarkers

Perhaps the greatest obstacle to clinical translation is the absence of robust biomarkers capable of reliably identifying senescent cells in human tumors. Current approaches commonly rely on combinations of p16INK4a, p21CIP1/WAF1, SA-β-gal activity, Lamin B1 loss, persistent DNA-damage signaling such as γH2AX, lipofuscin accumulation, SASP components, and/or senescence-associated transcriptional signatures. However, no single marker is universally expressed across all senescent cell types, and many commonly used markers can also be induced by cellular stress or other non-senescent states [3]. Recent consensus guidelines similarly emphasize that identification of senescence in vivo requires the assessment of multiple complementary features rather than reliance on an individual marker [175].

The development of standardized biomarker panels will therefore likely require multidimensional integration of transcriptomics, proteomics, metabolomics, epigenetic profiling, spatial transcriptomics, circulating biomarkers, and potentially molecular imaging. In particular, spatial approaches may be valuable because the biological effects of senescent cells depend strongly on their cellular neighbors and tissue localization. Single-cell approaches are also beginning to demonstrate that senescence encompasses multiple transcriptional states rather than a single homogeneous phenotype; for example, machine-learning analysis of 602 samples from 52 senescence transcriptomic datasets identified distinct senescence identities with different biological characteristics and responses to senolytics [176].

The identification of clinically applicable biomarkers capable not only of detecting senescent cells but also of distinguishing transient from persistent, immunologically cleared from immune-evasive, and therapeutically beneficial from potentially detrimental senescence therefore represents one of the highest priorities in the field.

### 9.3. Understanding Senescence Heterogeneity

Accumulating evidence demonstrates that senescence is not a uniform cellular state. Instead, multiple senescent subpopulations can coexist within tumors and their surrounding microenvironment, including malignant cells, cancer-associated fibroblasts, endothelial cells, macrophages, lymphocytes, and other stromal populations. These populations may exhibit distinct SASP compositions, metabolic profiles, immune interactions, survival mechanisms, and sensitivities to senolytic agents [3,177]. Recent single-cell analyses have provided direct evidence for substantial heterogeneity in senescence-associated transcriptional states and trajectories [176].

Similarly, different therapeutic modalities—including chemotherapy, radiotherapy, targeted therapy, endocrine therapy, and some immunotherapeutic interventions—can induce qualitatively different senescence programs. The resulting phenotype is influenced by the nature and intensity of the initiating stress, cellular lineage, genetic background, metabolic state, and duration of treatment [174]. Thus, TIS may encompass multiple biologically distinct states rather than a single conserved program.

A comprehensive molecular classification of TIS analogous, although not necessarily identical, to current molecular tumor classifications may therefore become necessary to guide personalized senotherapeutic approaches. Such classification could integrate the identity of the senescent cell, the inducing therapy, the dominant survival pathways, SASP composition, immune context, and susceptibility to specific senolytic or senomorphic interventions.

### 9.4. Optimizing Senolytic Therapy

Although senolytics represent one of the most promising translational applications of senescence biology, numerous questions remain regarding their optimal clinical implementation. The central challenge is that senescent cells are heterogeneous and may depend on distinct pro-survival mechanisms. Early work identified several SCAPs that protect senescent cells from apoptosis and consequently provide potential therapeutic vulnerabilities [154,178]. More recent studies have confirmed that senescent cells can display heterogeneous survival dependencies, suggesting that no single senolytic agent is likely to eliminate all senescent populations [177].

Key unanswered clinical questions therefore include:

- Which patients should receive senolytic therapy?
- What is the optimal interval between chemotherapy or radiotherapy and senolytic administration?
- Should senolytics be administered as a single pulse or repeated cycles?
- Which senescent populations should be preferentially eliminated?
- Can normal-tissue toxicity be minimized while maintaining antitumor efficacy?
- How should senolytic response and residual senescent-cell burden be monitored longitudinally?

Addressing these questions will require carefully designed biomarker-driven clinical trials rather than empirical combination therapies. This consideration is particularly important because the optimal treatment sequence may depend on the kinetics of senescence induction and the emergence of senescence-associated vulnerabilities. The “one–two punch” model proposes that a first treatment induces senescence and a subsequent senolytic intervention selectively eliminates the resulting senescent tumor cells [131,174].

Future senolytics may therefore need to be selected according to tumor genotype, treatment history, cellular lineage, and senescence phenotype rather than applied as universal agents. This approach could ultimately shift senolytic therapy from empiric drug administration toward phenotype-guided senolysis.

### 9.5. Integrating Senescence into Precision Oncology

Cancer therapy is increasingly guided by molecular profiling of tumors, including genomic, transcriptomic, proteomic, and immune-based characterization. However, senescence has not yet been routinely incorporated into precision oncology despite its potential influence on treatment response, residual disease, and tumor recurrence. Current evidence suggests that the biological consequences of senescence depend on the identity and abundance of senescent cells, their secretory phenotype, their interactions with immune populations, and their capacity to persist or escape growth arrest [3,174].

Including assessment of senescence burden, SASP composition, immune surveillance status, metabolic phenotype, and senescence-escape signatures could potentially identify patients most likely to benefit from senolytics, senomorphics, immune-mediated senescence clearance, or sequential “one–two punch” strategies. However, these biomarkers require prospective validation before they can be used routinely for treatment selection.

Integration of senescence biology with precision oncology therefore represents an important opportunity for personalized cancer treatment. A future precision-senescence framework could combine tumor genomic alterations with TIS profiles, spatial distribution of senescent cells, immune contexture, and predicted senolytic vulnerabilities to guide treatment selection and scheduling.

### 9.6. Artificial Intelligence and Systems Biology

The complexity and heterogeneity of senescence increasingly exceed the analytical capacity of conventional single-marker approaches [179]. Artificial intelligence (AI) and machine learning are therefore expected to become important tools for automated identification of senescent cells, prediction of TIS, characterization of senescence-associated transcriptional states, identification of therapeutic vulnerabilities, and patient stratification. An important proof of concept is provided by the SenCID machine-learning framework, which was trained on 602 samples from 52 senescence transcriptomic datasets spanning 30 cell types and identified multiple senescence identities with distinct biological properties and responses to senolytic interventions [176].

Similarly, systems biology approaches capable of modeling interactions among tumor cells, stromal cells, immune populations, and the ECM may improve understanding of the complex ecological networks established by persistent senescent cells. Integration of single-cell transcriptomics, spatial biology, proteomics, metabolomics, and computational network analysis may ultimately enable dynamic modeling of senescence within individual tumors.

Nevertheless, AI-derived senescence signatures will require extensive validation across independent cohorts, tissue types, treatment regimens, and clinical platforms. Standardized definitions of senescence and high-quality annotated datasets will be essential for ensuring that computational models distinguish genuine senescence from transient cellular stress or other states of cell-cycle arrest.

## 10. Conclusions

Cellular senescence has emerged as one of the most paradoxical biological responses in cancer therapy. Initially regarded primarily as an irreversible TIS mechanism, therapy-induced senescence is now recognized as a heterogeneous and dynamic cellular state whose long-term consequences depend on the balance between senescence induction, persistence, immune surveillance, and elimination [3,174]. While acute senescence can suppress tumor growth by establishing durable proliferative arrest and facilitating immune-mediated clearance, persistent senescent cells can remodel the TME through the SASP, alter cellular plasticity, influence CSC behavior, undergo metabolic and epigenetic reprogramming, and contribute to an inflammatory milieu that may support tumor progression [3,158,174].

Collectively, these mechanisms position TIS as an important determinant of minimal residual disease, tumor recurrence, and acquired resistance across multiple therapeutic modalities, including chemotherapy, radiotherapy, and targeted therapy. However, the extent to which TIS directly drives these clinical outcomes likely varies according to tumor type, therapeutic context, and host immune status. These observations therefore challenge the traditional view that induction of senescence alone is sufficient for durable therapeutic benefit and instead support a more nuanced model in which the biological outcome of senescence is determined by its temporal regulation, cellular heterogeneity, and interaction with the TME.

The rapid development of senolytic agents, senomorphic therapies, and immune-based strategies targeting senescent cells has opened new opportunities to exploit the beneficial effects of TIS while limiting its potentially detrimental consequences. Among these approaches, the sequential “one–two punch” paradigm exemplifies a rational therapeutic strategy in which senescence induction is followed by selective elimination of persistent senescent cells. The underlying rationale is to exploit vulnerabilities exposed by the senescent state while reducing the persistence of cells capable of producing tumor-supportive signals [131,174].

Although this concept has demonstrated considerable promise in preclinical models, successful clinical translation will require robust biomarkers, optimized treatment schedules, improved understanding of senescent-cell heterogeneity, and prospective biomarker-guided clinical trials. In particular, the development of standardized approaches for detecting and quantifying senescence in vivo remains a fundamental prerequisite. Recent Minimal Information on Cellular Senescence Experimentation in vivo (MICSE) recommendations emphasize the need for harmonized approaches to senescence assessment and acknowledge the limitations of currently available markers in human tissues [175].

Looking forward, advances in single-cell multi-omics, spatial biology, systems immunology, AI, and precision oncology may help address the central problem of senescence heterogeneity. Their most useful role in this context is not simply to classify cells as senescent, but to identify molecularly distinct senescent populations, distinguish senescence from related persister states, define clinically relevant biomarker combinations, and potentially guide patient selection and treatment timing. These approaches remain investigational and require independent biological and clinical validation before they can be used as actionable tools.

Rather than representing the terminal endpoint of the therapeutic response, TIS should therefore be regarded as a dynamic and potentially targetable phase of tumor evolution. Understanding and controlling this phase may ultimately become an important component of next-generation cancer therapy. The central translational challenge, however, is not simply to eliminate senescent cells, but to determine which senescent cells should be targeted, when they should be targeted, and whether their selective modulation produces a clinically meaningful improvement in tumor control and patient survival. Resolving these questions will determine whether senescence-targeted therapy progresses from a promising experimental concept to a clinically actionable component of precision oncology.

## 11. Review Scope and Evidence Appraisal

### 11.1. Review Methodology

This article was developed as a narrative and conceptual review of the current evidence concerning cellular senescence in cancer, with particular emphasis on therapy-induced senescence, treatment resistance, the senescence-associated secretory phenotype (SASP), senescence-associated biomarkers, and therapeutic strategies targeting senescent cancer cells and the tumor microenvironment. The literature was identified through searches of PubMed/MEDLINE, Scopus, and Web of Science, supplemented by examination of the reference lists of relevant publications to identify additional studies of particular relevance. The literature search considered publications available from January 2000 through July 2026. The principal search concepts included cellular senescence, therapy-induced senescence, cancer, tumor, chemotherapy, radiotherapy, targeted therapy, immunotherapy, treatment resistance, senescence-associated secretory phenotype, senescence biomarkers, senolytics, senomorphic agents, and senescent cells. These concepts were combined using Boolean operators, including combinations of (“cellular senescence” OR “therapy-induced senescence”) AND (cancer OR tumor) AND (treatment OR therapy OR chemotherapy OR radiotherapy OR immunotherapy) AND (resistance OR response OR outcome), with additional combinations adapted to specific thematic areas.

Literature was selected according to its relevance to the objectives and scope of the review. Particular emphasis was placed on peer-reviewed original experimental studies, translational studies, clinical studies, and selected review articles providing mechanistic, biological, therapeutic, or clinical information relevant to cancer-associated senescence and treatment response or resistance. Studies focused exclusively on senescence outside the context of cancer, publications that did not address the principal themes of the review, and articles lacking information relevant to the biological or clinical questions under consideration were not prioritized. Relevant publications identified through reference-list screening were also considered when they provided important mechanistic, translational, or clinical evidence not otherwise captured by the primary database searches.

The identified evidence was organized thematically rather than according to a formal systematic-review protocol. Preclinical/mechanistic and clinical evidence were considered separately whenever possible, recognizing that observations derived from experimental models may not directly translate to patients. In interpreting apparently conflicting findings, consideration was given to differences in tumor type, treatment modality, experimental model, senescence definition and biomarkers, cellular compartment examined, timing of sample collection, and clinical endpoint. Findings supported by independent clinical cohorts, multiple experimental approaches, or convergent evidence across different study types were given greater interpretive weight, whereas findings derived primarily from exploratory or preclinical studies were interpreted cautiously.

Because this work was conceived as a narrative and conceptual review rather than a systematic review or meta-analysis, it was not conducted according to PRISMA or another formal systematic-review framework. Accordingly, no formal risk-of-bias assessment or quantitative meta-analysis was undertaken. The purpose of the literature-selection process was to provide a transparent and balanced synthesis of the mechanistic, translational, and clinical evidence relevant to cancer-associated senescence and its potential relationship with treatment response, resistance, and therapeutic vulnerability. Conclusions were therefore qualified according to the nature and strength of the available evidence, with particular attention to distinguishing established clinical observations from mechanistic inference and predominantly preclinical findings.

### 11.2. Evidence Appraisal



**Evidence Category**

**Definition**

**Interpretation**
Mechanistic/experimentalCellular or animal experiments identifying pathways or phenotypes that may link senescence to treatment response or resistance.Supports biological plausibility; does not by itself establish clinical causality.Preclinical therapeuticExperimental intervention studies testing senolytic, senomorphic, immune-mediated, or sequential strategies.Supports proof-of-concept and informs translational development.Clinical associationObservational or biomarker studies relating senescence-associated features to treatment response, recurrence, or outcome.Association does not establish that senescence caused the clinical outcome.Prospective/interventional clinicalProspective studies evaluating a senescence-related biomarker or intervention with predefined endpoints.Higher translational relevance; interpretation depends on study design and validation.Validated/actionableEvidence independently validated sufficiently to support routine clinical decision-making.Currently limited for senescence-directed treatment and biomarker-guided timing.


## Figures and Tables

**Figure 1:**
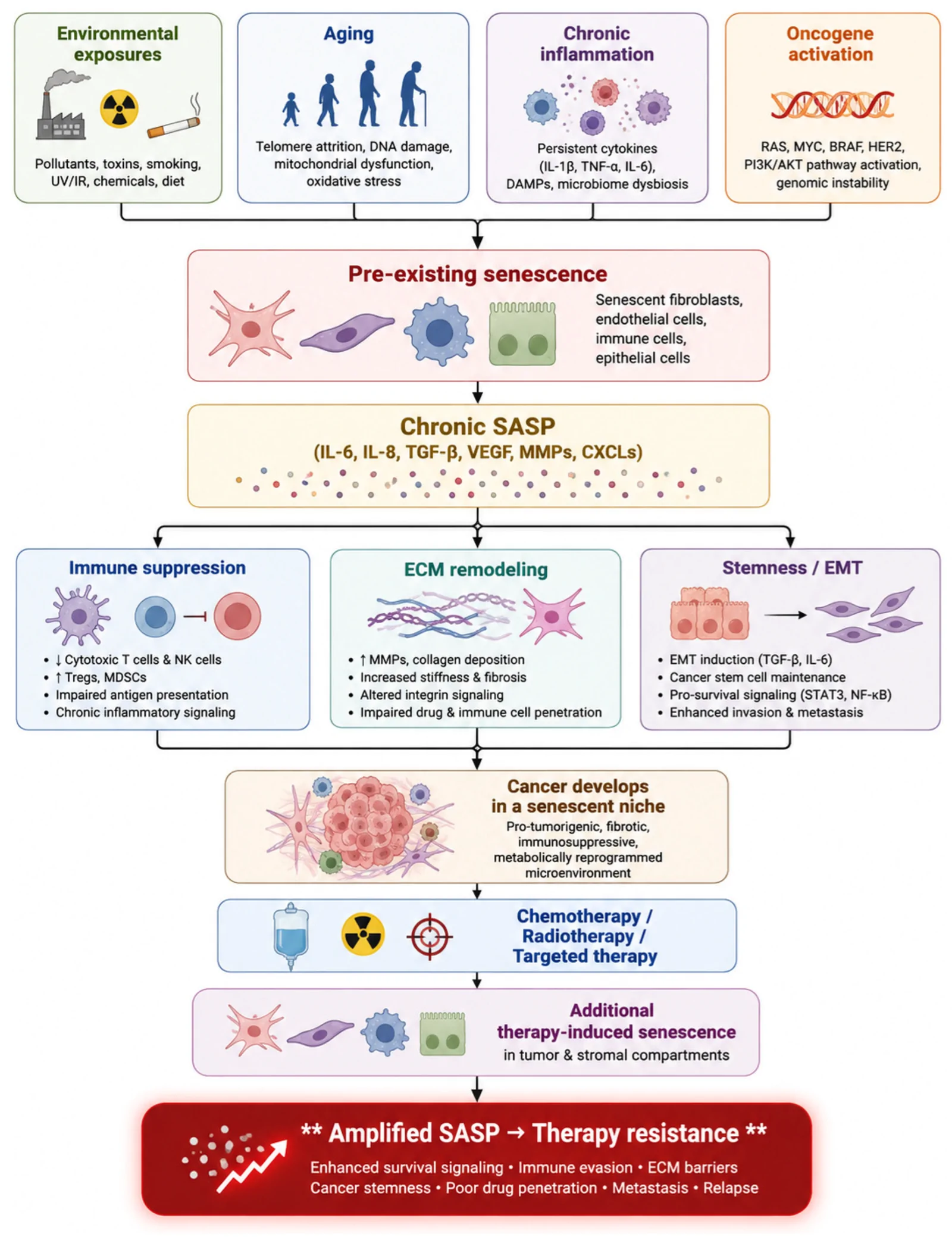
Overview of Senescence-induced Resistance to Cancer therapy. How Aging, Environmental Stress, and Carcinogenesis Shape a Therapy-Resistant Tumor Microenvironment through cellular senescence and SASP induction.

**Figure 2:**
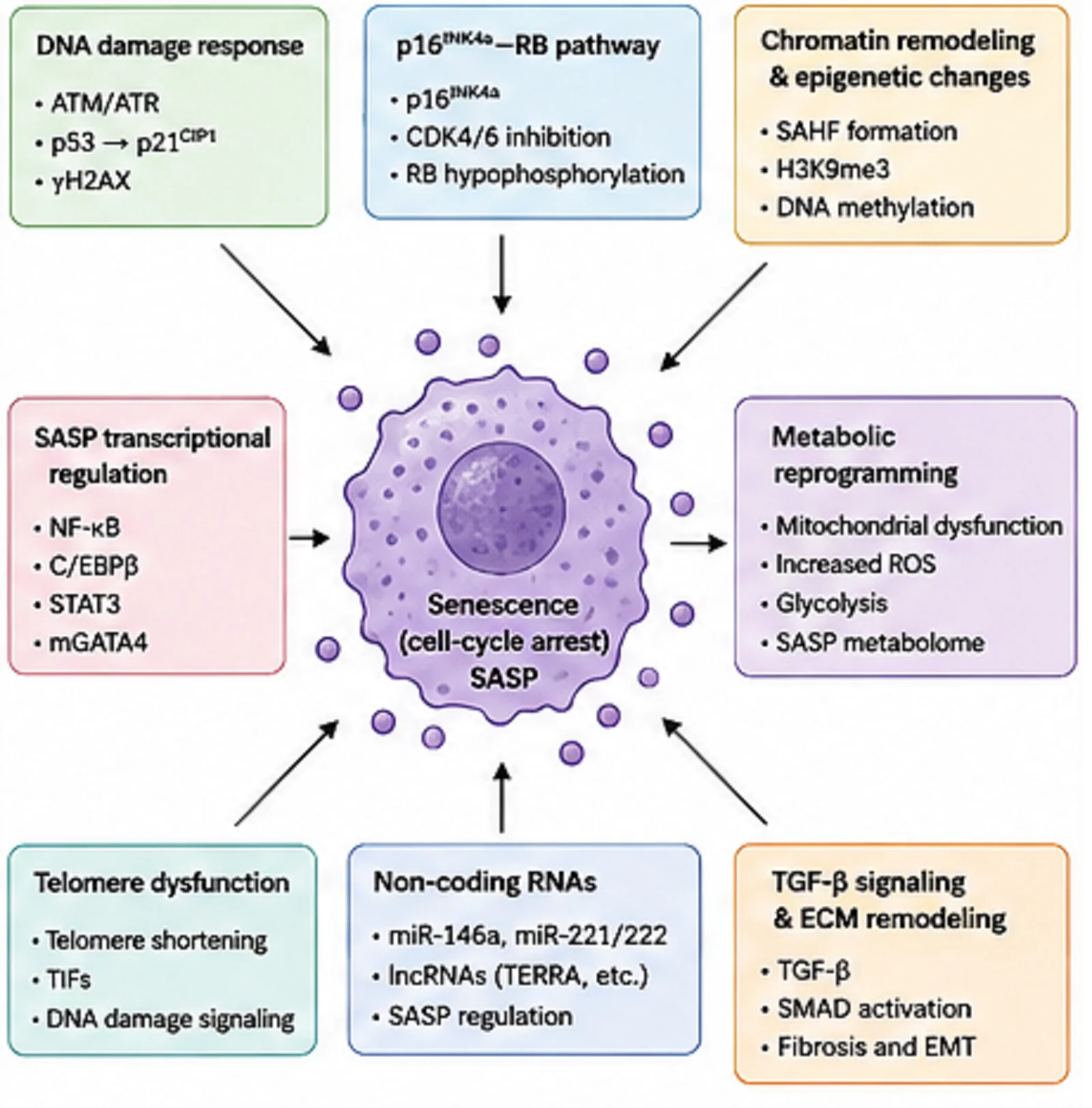
Main molecular pathways involved in Senescence induction.

**Figure 3:**
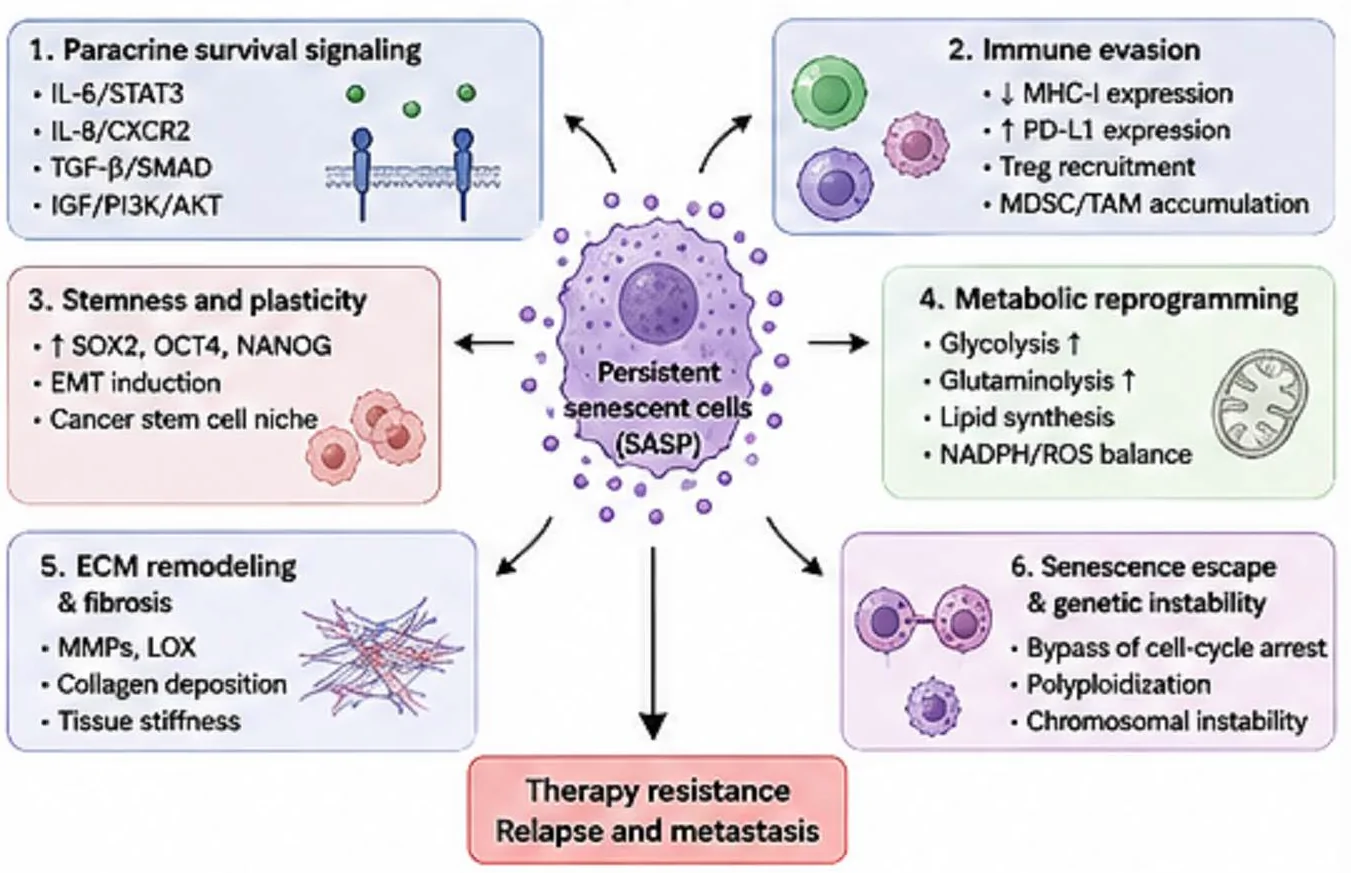
Mechanisms by which persistent senescent cells promote therapy resistance.

**Figure 4:**
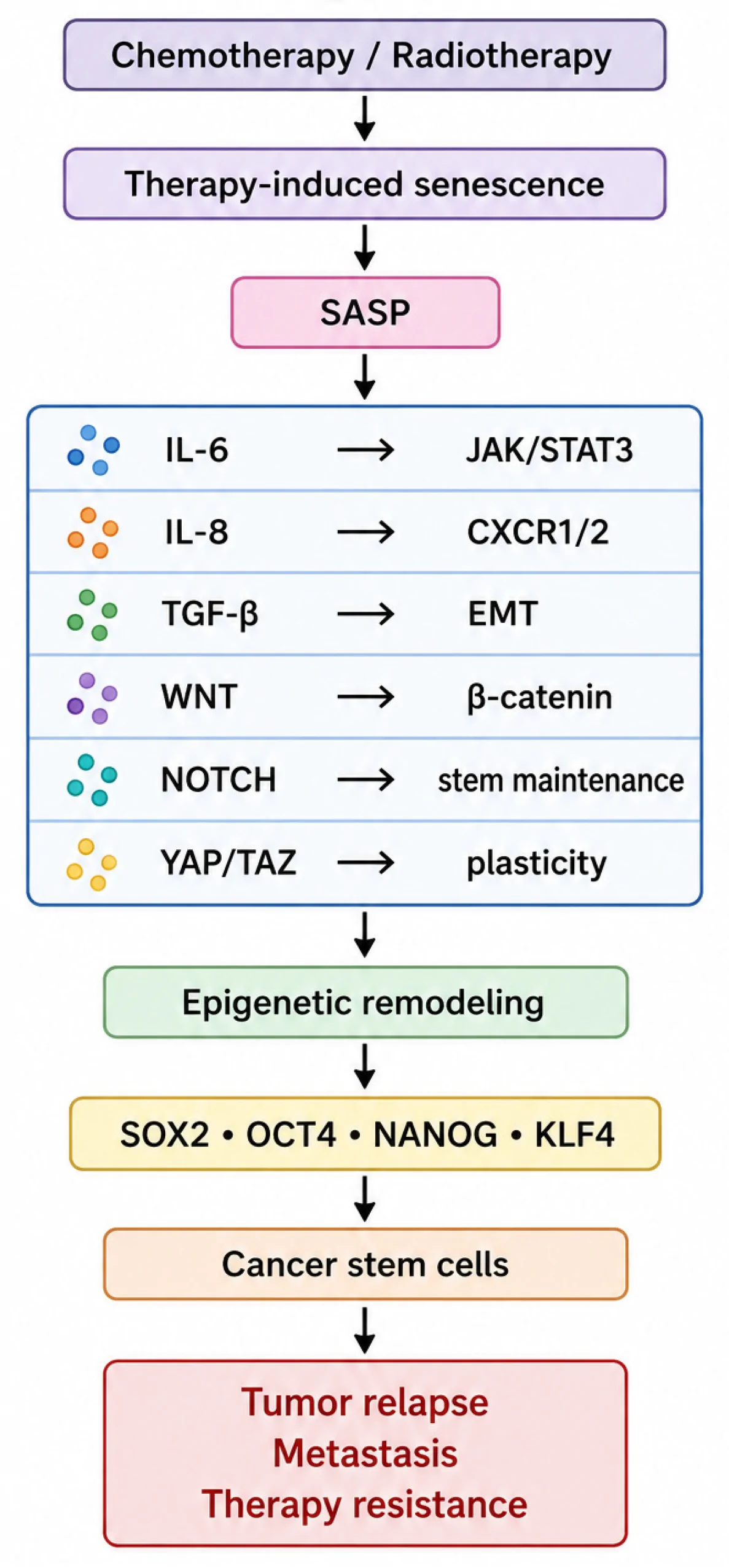
Scheme showing the possible mechanism through therapy-Induced Senescence Promotes Cellular Plasticity and Cancer Stemness.

**Figure 5:**
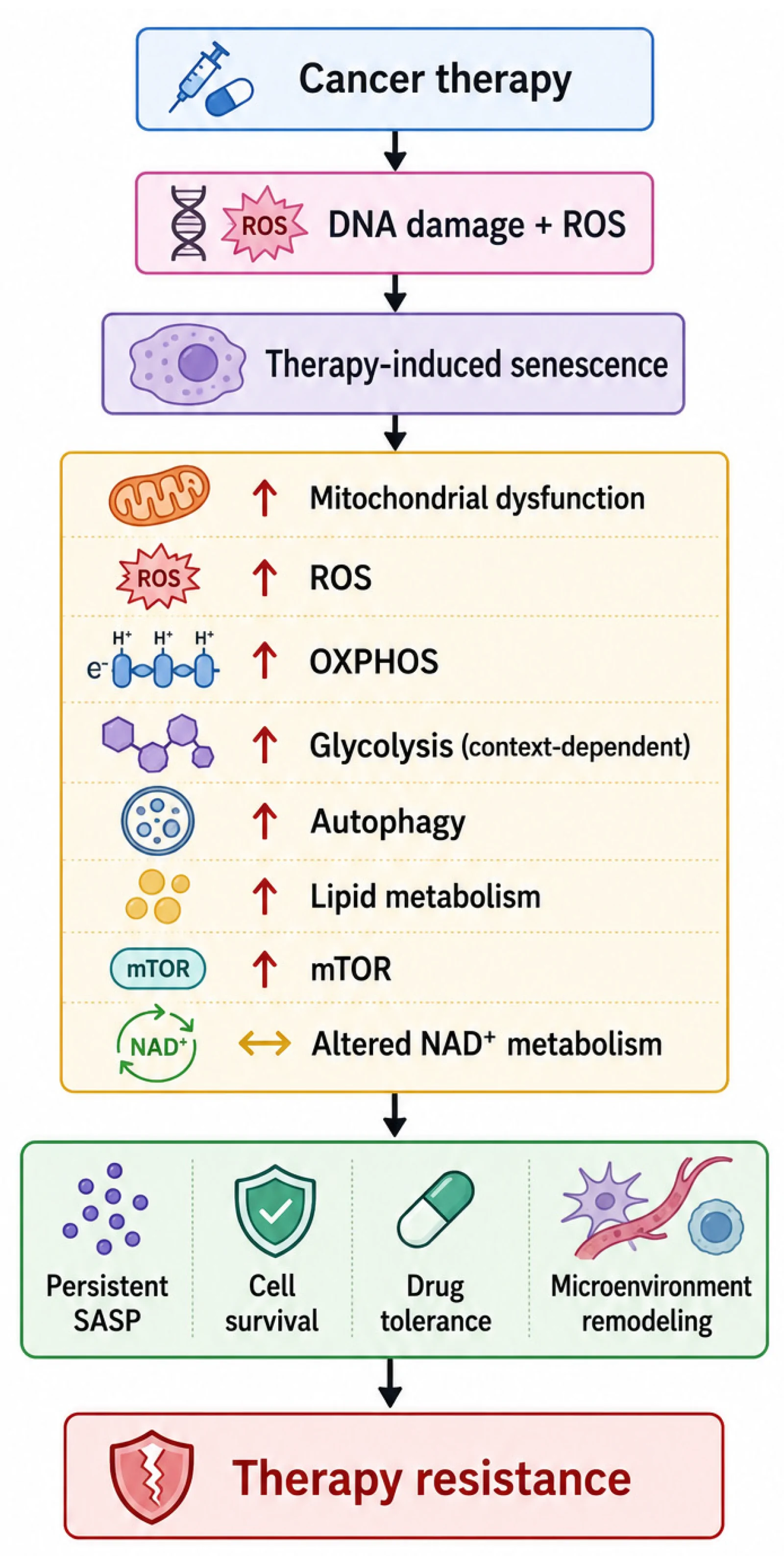
Metabolic hallmarks of therapy-induced senescence.

**Figure 6:**
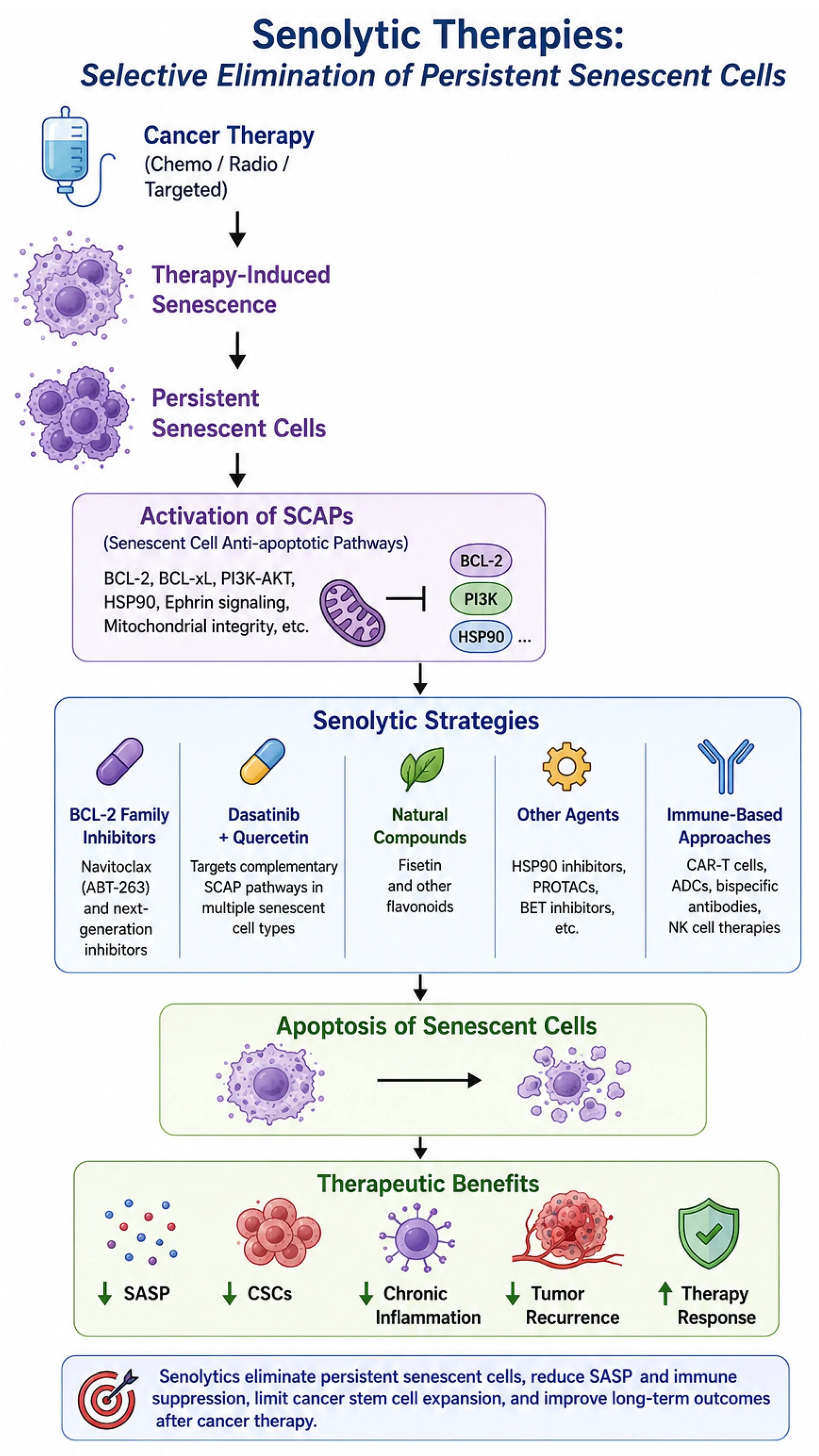
Senolytic therapy: Selective elimination of persistent senescent cells.

**Figure 7:**
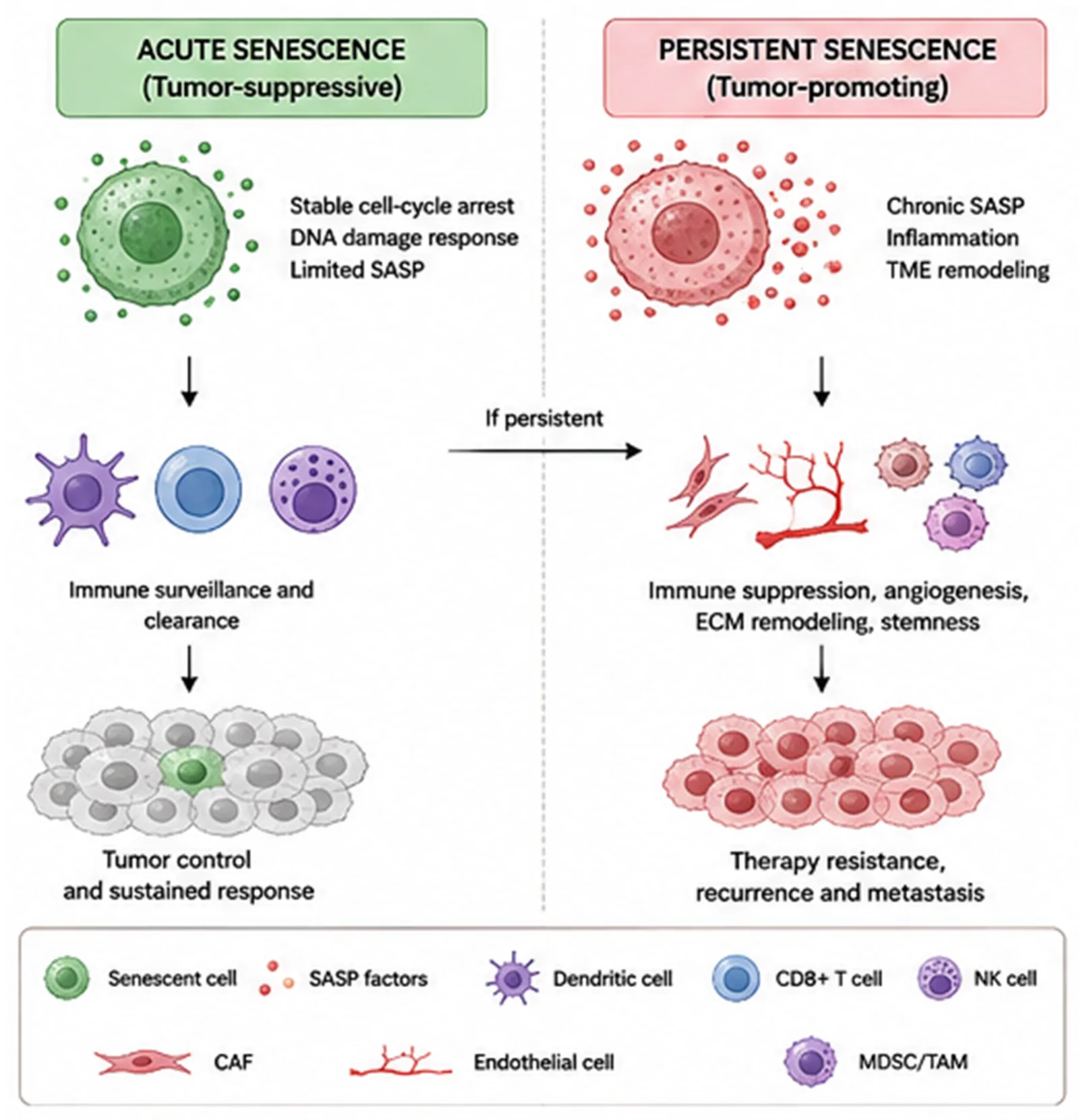
The “One–Two Punch” therapeutic paradigm: The double nature of Therapy-induced senescence in cancer.

**Table 2:** Clinical evidence linking therapy-induced senescence with therapeutic resistance.

Therapy Modality	Principal Stress	Senescence-Associated Response	Potential Resistance Mechanism	Potential Intervention	Evidence Status
Chemotherapy	DNA damage, replication stress, oxidative stress	Durable arrest, DDR activation, SASP, metabolic remodeling	Persistent SASP, plasticity/stemness, immune evasion, residual-cell survival	Senolytic or senomorphic treatment after selected regimens	Strong preclinical; heterogeneous clinical evidence
Radiotherapy	DNA damage and persistent DDR	Persistent arrest, inflammatory signaling, stromal effects	Microenvironment remodeling, fibrosis, immune modulation, persistence	Senolytic/senomorphic or immune-mediated clearance	Strong preclinical; limited clinical validation
Targeted therapy	Oncogene/pathway inhibition and cellular stress	Context-dependent senescence or senescence-like persister states	Phenotypic plasticity and adaptive persistence	Context-specific senescence targeting	Mechanistic and preclinical; selected clinical associations
Immunotherapy	Immune-mediated stress and treatment-associated cellular responses	Senescence in tumor/stromal compartments; senescence-like immune phenotypes	Immune evasion or altered surveillance	Immune-mediated clearance; combinations under investigation	Emerging; substantial mechanistic uncertainty

**Table 3:** Major senomorphic agents under investigation.

Agent	Primary Target	Principal Effect on Senescence	Potential Benefit in Cancer
Rapamycin	mTOR	↓ SASP translation	Reduces inflammation/stromal Remodeling
Ruxolitinib	JAK1/2	↓ IL-6/STAT3 signaling	Limits CSC induction and chronic inflammation
Metformin	AMPK/mTOR	Improves mitochondrial homeostasis; ↓ SASP	Reduces metabolic adaptation
p38 MAPK	p38 MAPK inhibitors	↓ NF-κB activation	Suppresses inflammatory secretome
NF-κB inhibitors	NF-κB	Broad suppression of SASP genes	Decreases microenvironment Remodeling
Experimental cGAS-STING	cGAS-STING	Context-dependent regulation modulators of inflammatory	Fine-tunes immune surveillance versus chronic inflammation

**Table 4:** Selected Senescence-Targeted Therapeutic Strategies in Clinical and Preclinical Development.

Therapeutic Strategy	Representative Agents	Development Stage	Primary Limitations
BCL-2/BCL-xL inhibition (Senolytics)	Navitoclax (ABT-263)	Clinical (oncology)	Dose-limiting thrombocytopenia due to platelet dependence on BCL-xL
Tyrosine kinase inhibitor + flavonoid (Senolytics)	Dasatinib + Quercetin (D+Q)	Early clinical; preclinical oncology	Variable senolytic efficacy across different cell types and tissues
Natural senolytics	Fisetin	Early clinical (aging); preclinical (oncology)	Limited clinical evidence in cancer and uncertain optimal dosing
mTOR inhibition (Senomorphics)	Rapamycin, Everolimus	Clinical	Primarily suppresses SASP rather than eliminating senescent cells
JAK/STAT pathway inhibition (Senomorphics)	Ruxolitinib	Clinical	Broad immunomodulatory effects may impair anti-tumor immunity
Immune-mediated senolytics	uPAR CAR-T cells, NK cell–based therapies	Preclinical/Early translational	Safety, target specificity, and manufacturing complexity
PROTAC-based senolytics	BCL-xL PROTAC degraders	Preclinical	Limited in vivo validation and pharmacokinetic optimization required
Nanoparticle-guided senolytics	Experimental nanoparticle formulations	Preclinical	Targeted delivery, biodistribution, and long-term safety remain to be optimized

Abbreviations: BCL, B-cell lymphoma protein; CAR-T, chimeric antigen receptor T cells; D+Q, dasatinib plus quercetin; JAK, Janus kinase; mTOR, mechanistic target of rapamycin; NK, natural killer; PROTAC, proteolysis-targeting chimera; SASP, senescence-associated secretory phenotype; uPAR, urokinase plasminogen activator receptor.

## Data Availability

No new data were created or analyzed in this study. Data sharing is not applicable to this article.

## Supplementary Materials

The following supporting information can be downloaded at https://www.mdpi.com/article/10.3390/cancers18193084/s1. Table S1: Clinical and translational evidence linking cancer-associated senescence with treatment response, resistance, and clinical outcomes.
